# A Zinc Finger Protein-Based Prognostic Model in Lung Adenocarcinoma Identifies FGD3 as a Marker Associated with Lorlatinib Resistance

**DOI:** 10.3390/cancers18101591

**Published:** 2026-05-14

**Authors:** Jiayue Sun, Yue Yang, Xiaoyi Huang, Dinglong Xue, Jiazhuang Li, Yaru Huang, Qingwei Meng

**Affiliations:** 1 Department of Medical Oncology, Harbin Medical University Cancer Hospital, No. 150, Haping Road, Harbin 150040, China; sunjiayue163@163.com (J.S.); yangyue4585@163.com (Y.Y.); 2020021658@hrbmu.edu.cn (D.X.); lijiazhuang1988@163.com (J.L.); 2019021567@hrbmu.edu.cn (Y.H.); 2 Biotherapy Center, Harbin Medical University Cancer Hospital, Harbin 150081, China; xyhuang@hrbmu.edu.cn; 3 NHC Key Laboratory of Cell Transplantation, Harbin Medical University, Harbin 150081, China

**Keywords:** lung adenocarcinoma, zinc finger proteins, prognostic model, FGD3, lorlatinib resistance

## Abstract

**Background**: Lung adenocarcinoma (LUAD) is the most common type of lung cancer and a major cause of cancer death. Zinc finger proteins (ZNFs) have been implicated in LUAD progression, functioning either as oncogenes or tumor suppressors. Therefore, an in-depth investigation of ZNFs may contribute to the development of novel diagnostic and therapeutic strategies for LUAD. **Methods**: Transcriptomic and clinical data were obtained from the TCGA and GEO databases. Prognosis-related ZNF genes were identified using univariate Cox, LASSO, and multivariate Cox regression analyses. An eight-gene ZNF-based prognostic signature was constructed and validated in two independent external cohorts (GSE50081 and GSE26939). A nomogram integrating independent prognostic factors was developed. Immune infiltration, somatic mutation profiles, and drug sensitivity were systematically analyzed. We further focused on FGD3, a key gene from the signature, examining its expression in LUAD cells and tissues, including lorlatinib-resistant models. **Results**: The prognostic signature comprising TRIM6, TRIM29, CTCFL, FGD3, GATA4, CASZ1, TRAF2, and ZNF322 effectively stratified patients into distinct risk groups with significantly different overall survival (*p* < 0.05). The risk score, together with T and N stage, served as independent prognostic predictors (*n* = 500, *p* < 0.05). High-risk patients exhibited an immune-desert phenotype, increased tumor mutational burden, and distinct drug sensitivity patterns. Notably, FGD3 expression was downregulated in LUAD tissues (*n* = 14, *p* < 0.0001) and lorlatinib-resistant cells, and its restoration suppressed resistant cell proliferation and partially reversed drug resistance. **Conclusions**: This study establishes a promising ZNF-based prognostic model for LUAD, providing a potential tool for risk stratification and individualized therapeutic decision-making. The identification of FGD3 as a potential mediator of drug resistance highlights its promise as a candidate biomarker and therapeutic target in LUAD.

## Simple Summary

Lung adenocarcinoma, the most common lung cancer, is driven by disruptions in gene regulation. This study focused on zinc finger proteins, the largest family of gene regulators in humans, to develop a more accurate tool for predicting patient outcomes. Using public patient data, we built a prognostic model based on eight zinc finger proteins and validated it in independent patient groups. The model successfully distinguished low-risk from high-risk patients with significantly different survival outcomes. We further identified one of these proteins, FGD3, which is present at low levels in lung adenocarcinoma. Lower FGD3 expression was linked to reduced sensitivity to ALK inhibitor drugs. When we increased FGD3 expression in drug-resistant cancer cells, their growth and spread were suppressed, and their sensitivity to the ALK inhibitor lorlatinib was partially restored. These findings offer a promising approach for personalizing lung cancer treatment and provide new insights into overcoming drug resistance.

## 1. Introduction

Lung cancer is the most commonly diagnosed cancer worldwide, with approximately 2.5 million new cases each year, accounting for 12.4% of all cancers. It also ranks first in both incidence and mortality among cancers in China [1]. Non-small-cell lung cancer (NSCLC) accounts for 80–85% of cases, with lung adenocarcinoma (LUAD) being the most prevalent subtype [2]. Although most patients initially respond to standard chemotherapy, relapse frequently occurs [3]. LUAD exhibits high heterogeneity, driven by genetic alterations such as EGFR, KRAS, and ALK mutations, which serve as major targets for molecular therapy [4]. For patients without actionable mutations, immune checkpoint inhibitors (ICIs) provide a promising option [3]. Despite therapeutic advances, the prognosis for advanced LUAD remains poor due to limited prognostic markers. Biomarkers such as TP53 mutations, PD-L1 expression, immune microenvironment features, and tumor metabolism-related molecules have been used to predict outcomes [5]; however, their clinical utility remains limited due to inconsistent predictive performance across patient cohorts and insufficient robustness in capturing tumor heterogeneity [6]. Most existing biomarkers rely on single-gene or single-pathway features, which are inherently inadequate to reflect the complex, multi-layered regulatory landscape of LUAD. This highlights the urgent need for more integrative and system-level prognostic strategies.

Transcriptional dysregulation is a core feature of tumor initiation and progression [7]. Zinc finger proteins (ZNFs) constitute the largest family of transcription factors in the human genome and represent a key component of the transcriptional regulatory network, widely participating in chromatin remodeling, gene transcription, cell fate determination, and tumorigenesis [8]. These proteins contain one or more zinc ions coordinated with specific amino acids, forming diverse zinc-finger domains [9]. This structural versatility enables ZNFs to interact with DNA, RNA, and proteins, thereby regulating numerous biological processes, including transcription, DNA repair, protein degradation, signal transduction, cell proliferation, adhesion, migration, and cytoskeletal organization [10]. Here, we adopt an expanded structural definition of the ZNF family, encompassing all proteins containing zinc-finger domains (including C2H2, RING, PHD, FYVE, and other types), rather than limiting the definition to classical C2H2-type zinc finger transcription factors. Emerging evidence suggests that ZNFs can function either as oncogenes or tumor suppressors, depending on the cellular context [8]. For example, ZNF131 [11] and ZNF687 [12] have been reported to promote LUAD proliferation, invasion, and stemness, whereas ZNF652 acts as a tumor suppressor by downregulating cyclin D3 [13]. Similarly, CBX4 promotes LUAD cell proliferation while inhibiting metastasis [14].

Notably, ZNFs are a double-edged sword that can play dual roles in cancer, which makes any single ZNF unreliable as a standalone biomarker. Therefore, developing a multi-ZNF-signature-related model is a logical strategy to capture more comprehensive and reliable prognostic information, providing a complementary tool for risk stratification and individualized therapy.

In this study, we systematically investigated the expression and prognostic relevance of ZNFs in LUAD. Transcriptomic and clinical data were obtained from The Cancer Genome Atlas (TCGA), and ZNFs were retrieved from the UniProt database. Differentially expressed ZNFs (DE-ZNFs) were identified, and TCGA data were used to construct a prognostic model, which was subsequently validated in an independent GEO cohort. The model’s prognostic performance, immune landscape, mutation profiles, and drug sensitivity were further evaluated. This work provides new insights into the role of ZNFs in LUAD and may inform the development of individualized therapeutic strategies.

## 2. Materials and Methods

### 2.1. Data Acquisition

RNA sequencing data and corresponding clinical information for LUAD were obtained from the TCGA database https://portal.gdc.cancer.gov/ (accessed on 16 November 2024) using the TCGAbiolinks package [15], comprising 513 LUAD and 58 normal lung tissue samples. External validation cohorts were derived from the Gene Expression Omnibus (GEO) database http://www.ncbi.nlm.nih.gov/geo (accessed on 14 February 2025), specifically from GSE50081 (127 LUAD samples) [16] and GSE26939 (116 LUAD samples) [17]. Additionally, two GEO datasets containing paired LUAD tumor and adjacent normal tissues, GSE32863 (58 paired samples) [18] and GSE75037 (83 paired samples) [19], were used for external validation of FGD3 expression differences. All GEO datasets were normalized using the robust multi-array average method implemented in the limma package [20]. Annotations GPL570, GPL9053 and GPL6884 were used for ID conversion. Probes without gene symbols were excluded, and for multiple probes mapping to the same gene, the mean expression value was retained. Genes belonging to the zinc finger protein family (Supplementary Table S1) were retrieved from the Universal Protein Resource (UniProt) database https://www.uniprot.org/ (accessed on 14 February 2025).

### 2.2. Identification of Differentially Expressed ZNF Genes in LUAD

Differential expression analysis of the TCGA count data from LUAD tumors and adjacent normal tissue samples was performed using the DESeq2 package (version 1.36.0; Bioconductor, Boston, MA, USA) in R. Genes meeting the criteria of |log_2_ fold change| > 1 and adjusted *p* < 0.05 after Benjamini–Hochberg correction were selected. By intersecting these genes with the previously obtained ZNF gene set, differentially expressed ZNFs (DE-ZNFs) were identified. Heatmaps were generated using the pheatmap package (CRAN, Vienna, Austria), and volcano plots were constructed with the ggplot2 package (CRAN, Vienna, Austria).

### 2.3. Functional Enrichment Analysis

Gene Ontology (GO), Kyoto Encyclopedia of Genes and Genomes (KEGG), and Gene Set Enrichment Analysis (GSEA) were performed using the clusterProfiler package (Bioconductor, Boston, MA, USA) [21]. The MSigDB Hallmark gene set collection version 7.0 (Broad Institute, Cambridge, MA, USA) was used for GSEA. For GO and KEGG analyses, *p* < 0.05 was set as the threshold for statistical significance. For GSEA, gene sets with a false discovery rate (FDR) < 0.25 and *p* < 0.05 were considered significantly enriched. The results of the enrichment analyses were visualized using the ggplot2 and ggnewscale (CRAN, Vienna, Austria) packages.

### 2.4. Risk Prognostic Model Based on DE-ZNFs

Using TCGA as the training set, univariate Cox regression analysis was performed on DE-ZNFs with the survival package (CRAN, Vienna, Austria), using overall survival (OS) as the evaluation criterion. Genes significantly associated with OS (*p* < 0.05) were retained. Subsequently, least absolute shrinkage and selection operator (LASSO) regression was conducted using the glmnet (CRAN, Vienna, Austria) and survival packages, with 10-fold cross-validation to select the optimal lambda value. To avoid overfitting, stepwise Cox regression (direction = “both”) was applied. A random seed of 1 was used before LASSO cross-validation to ensure reproducibility. The risk score for the model was calculated as follows: Riskscore=β1E1+β2E2+…+βiEi, where *β* represents the regression coefficient, *E* denotes the expression level of each prognostic gene, and *i* is the number of genes. LUAD patients were stratified into high- and low-risk groups based on the median risk score. Kaplan–Meier survival curves were generated, and log-rank tests were conducted to evaluate survival differences between groups. The model’s predictive performance was assessed using receiver operating characteristic (ROC) curve analysis, with the area under the curve (AUC) representing its discriminatory ability. Time-dependent ROC analysis was performed using the timeROC package (CRAN, Vienna, Austria).

To validate the model in external GEO cohorts (GSE50081 [16] and GSE26939 [17]), the TCGA-derived risk score formula was applied. GEO data were log_2_-transformed to match the distribution of TCGA log_2_ (TPM + 1) data. Risk scores were calculated using the same Cox coefficients. Before calculation, expression of the eight genes in each GEO cohort was z-score standardized. High- and low-risk groups were defined using the cohort-specific median risk score as the cutoff.

### 2.5. Immune Cell Infiltration Analysis

CIBERSORT analysis was performed using the IOBR package (CRAN, Vienna, Austria) to compare the relative abundance of 22 immune cell types between the high- and low-risk groups [22], with 1000 permutations for robust estimation. Single-sample gene set enrichment analysis (ssGSEA) was performed with the GSVA package (Bioconductor, Boston, MA, USA) to evaluate differences in 28 immune cell types [23]. xCell analysis was performed using the xCell package (CRAN, Vienna, Austria) to quantify 64 immune cell types [24]. Correlation heatmaps between the eight model genes and the 22 CIBERSORT-derived immune cell types were generated using the corrplot (CRAN, Vienna, Austria) and ggcorrplot (CRAN, Vienna, Austria) packages. The estimate package (CRAN, Vienna, Austria) was used to calculate stromal, immune, and ESTIMATE scores, as well as tumor purity. Pearson’s correlation was used to assess associations between the risk score and these scores.

### 2.6. Prediction of Immunotherapy Response

LUAD data were downloaded from the TIDE database http://tide.dfci.harvard.edu/ (accessed on 28 February 2025) for tumor immune dysfunction and exclusion (TIDE) analysis [25]. A higher TIDE score indicates a poorer immunotherapy response. The immunophenoscores (IPS) were obtained from the Cancer Immunome Atlas https://tcia.at/ (accessed on 6 March 2025) and compared between risk groups. Unlike the TIDE score, a higher IPS is associated with a better predicted response to PD-L1/CTLA-4-targeted therapies [26]. Immunotherapy data from the urothelial carcinoma IMvigor210 dataset were also used to predict responses [27].

### 2.7. Tumor Mutation Burden and Chromosomal Location of Model Genes

The tumor mutation burden (TMB) data for the TCGA cohort were downloaded from the cBioPortal database https://www.cbioportal.org/ (accessed on 10 March 2025) and calculated using the Maftools package (Bioconductor, Boston, MA, USA) [28]; TMB was defined as the total number of non-synonymous mutations per megabase. Waterfall plots were generated for the top 15 mutated genes in each risk group. Violin and bar plots were created using the ggpubr package (CRAN, Vienna, Austria), and group comparisons were performed using chi-square tests. The rtracklayer (Bioconductor, Boston, MA, USA), RCircos (CRAN, Vienna, Austria), and magrittr (CRAN, Vienna, Austria) packages were used to plot copy number variation (CNV) landscapes, showing chromosomal locations of the DE-ZNF model genes.

### 2.8. Differential Gene Enrichment Analysis Between High- and Low-Risk Groups

Differentially expressed genes (DEGs) between the high- and low-risk groups were identified using the DESeq2 package with the Wald test. DEGs were defined as those with |log_2_ fold change| > 1 and *p* < 0.05. GO, KEGG, and GSEA of DEGs were performed using the clusterProfiler package. Gene set variation analysis (GSVA) was performed using the GSVA package [23].

### 2.9. Prediction of Antitumor Drug Sensitivity

Drug sensitivity data were obtained from the Genomics of Drug Sensitivity in Cancer (GDSC) and the Cancer Therapeutics Response Portal (CTRP). GDSC1, GDSC2, and CTRP2 data were downloaded from the GDSC website https://www.cancerrxgene.org/ (accessed on 17 April 2025) and the CTRP website http://portals.broadinstitute.org/ctrp.v2.1/ (accessed on 17 April 2025). The oncoPredict package (CRAN, Vienna, Austria) was used to predict drug sensitivity for high- and low-risk patients [29]. The half-maximal inhibitory concentration (IC_50_) values were estimated using ridge regression, expressed in µM, and log-transformed for downstream analyses.

### 2.10. Nomogram Construction

To evaluate the association between clinical characteristics and risk grouping, univariate and multivariate Cox regression analyses were performed to determine whether the clinical variables and the risk score were independent prognostic factors for LUAD patients. Clinical data for LUAD patients in the TCGA cohort were obtained from the UCSC Xena platform https://xenabrowser.net/datapages/ (accessed on 16 November 2024). Fisher’s exact test was applied to compare clinical characteristics between high- and low-risk groups using the tableone package (CRAN, Vienna, Austria). A nomogram was then constructed to enhance the prognostic accuracy and predictive power of the model [30]. The risk score, T stage, and N stage were identified as independent prognostic variables to estimate the probability of OS at 1, 2, and 3 years. Calibration curves were generated to assess the agreement between predicted and observed outcomes, thereby evaluating the nomogram’s predictive performance.

### 2.11. Validation of Prognostic Gene Expression

Expression levels of the prognostic genes were validated using the TCGA LUAD dataset. Bar plots were generated with ggpubr and ggsci (CRAN, Vienna, Austria), and group comparisons were performed using the Wilcoxon test. Immunohistochemical (IHC) data for the prognostic genes in LUAD tissues were obtained from the Human Protein Atlas (HPA) https://www.proteinatlas.org/ (accessed on 28 March 2025). ROC curves for the prognostic model genes were constructed using the pROC package (CRAN, Vienna, Austria) to evaluate their discriminatory performance.

### 2.12. Cell Culture and Clinical Specimen Collection

LUAD cell lines (H3122, H2228, H1650, H1993, and PC9) were purchased from the Cell Bank of the Chinese Academy of Sciences (Shanghai, China). RRID: CVCL_4974 (H3122), CVCL_1548 (H2228), CVCL_1483 (H1650), CVCL_1516 (H1993), CVCL_2144 (PC9). Human bronchial epithelial (HBE) cells were kindly provided by the Heilongjiang Cancer Institute (Harbin, China). RRID: CVCL_3568 (HBE). All cell lines were authenticated by short tandem repeat (STR) analysis and tested negative for mycoplasma. H3122, H1650, H2228, and PC9 cells were cultured in RPMI 1640 medium (Biological Industries, Beit HaEmek, Israel, Cat#01-100-1A) supplemented with 10% fetal bovine serum (FBS) (Biological Industries, Beit HaEmek, Israel, Cat#04-121-1B) and 1% penicillin–streptomycin (Beyotime, Shanghai, China, Cat#C0222). H1993 cells were maintained in DMEM (Biological Industries, Beit HaEmek, Israel, Cat#01-052-1A). All cells were incubated at 37 °C in a humidified atmosphere containing 5% CO_2_. 

A total of 12 paired fresh-frozen LUAD tumor and adjacent normal tissues were collected for protein extraction. Additionally, 14 paired paraffin-embedded sections were obtained for IHC. All specimens were pathologically confirmed, and no patients had received preoperative therapy.

### 2.13. Western Blot Analysis

Cells and tissues were harvested and lysed on ice for 30 min using lysis buffer (Boster, Wuhan, China; cat# AR0102-100) supplemented with protease inhibitors (Boster, Wuhan, China; cat# AR1192). The lysates were centrifuged at 12,000 rpm for 15 min at 4 °C, and the supernatants were collected. Protein concentration was determined using a protein quantification kit (Beyotime, Shanghai, China; cat# P0012S). Equal amounts of protein (25 μg) were separated by SDS-PAGE and transferred to PVDF membranes (Merck/Amersham, Darmstadt, Germany; cat# 73493400/A30812848). Membranes were blocked with 5% nonfat milk overnight at 4 °C and incubated with primary antibodies for 24 h at 4 °C, followed by HRP-conjugated secondary antibodies for 50 min at room temperature. Immunoreactivity was detected using an ECL kit (TianNeng Technology, Shanghai, China; cat# 180-506; RRID: SCR_015160). Band intensities were quantified using ImageJ (version 1.54g; NIH, Bethesda, MD, USA; RRID: SCR_003070) and normalized to GAPDH. The primary antibodies used were rabbit anti-FGD3 (Proteintech, Wuhan, China; cat# 20347-1-AP; 1:1000; RRID: AB_2881315) and mouse anti-GAPDH (Proteintech, Wuhan, China; cat# 60004-1-Ig; 1:50,000; RRID: AB_2107436). HRP-conjugated secondary antibodies were purchased from Cell Signaling Technology (Danvers, MA, USA; cat# 7074 and 7076; RRID: AB_2099233 and AB_330924). Western blot experiments were performed independently at least three times (*n* = 3). Representative blots are shown. Quantitative data are presented as mean ± SD. 

### 2.14. IC_50_ Assay

Lorlatinib was purchased from MedChemExpress (Monmouth Junction, NJ, USA; cat# HY-15728). Cells (5000/well) were seeded in 96-well plates and cultured for 12 h. After adhesion, the medium was replaced with complete medium containing serial dilutions of lorlatinib (0–10 µM). After 48 h, Cell Counting Kit-8 (CCK-8, Sevenbio, Beijing, China; cat# SC11901) reagent was added, and plates were incubated for 1 h at 37 °C. Absorbance was measured at 450 nm. Dose-response curves were generated, and IC_50_ values were calculated using nonlinear regression in GraphPad Prism.

### 2.15. Lorlatinib-Resistant Cells

Parental H3122 cells were exposed to escalating doses of lorlatinib starting at 0.04 µM. The concentration was doubled stepwise until 1 µM was reached. The resulting cells exhibited an IC_50_ value 10 times higher than that of the parental cells. This resistant cell line was designated as H3122 lorlatinib-resistant cells (H3122LR). H3122LR cells were subsequently expanded and continuously maintained in medium containing the same lorlatinib concentration during each passage.

### 2.16. Immunohistochemistry

Immunohistochemistry (IHC) was performed using an IHC kit (Elabscience, Wuhan, China; cat# E-IR-R220). Briefly, paraffin-embedded sections were baked at 65 °C for 60 min, deparaffinized, and subjected to antigen retrieval for 30 min, followed by cooling to room temperature. Sections were sequentially incubated with peroxidase blocking buffer for 15 min and primary antibody overnight at 4 °C. After washing, sections were incubated with secondary antibody at room temperature for 30 min, followed by 3,3’-diaminobenzidine (DAB) staining for 3 min and hematoxylin counterstaining for 30 s. Sections were dehydrated through graded ethanol, cleared with xylene, mounted with neutral resin, and air-dried. The primary antibodies antibody used for IHC were was rabbit anti-FGD3 (Proteintech, Wuhan, China; cat# 20347-1-AP; 1:100; RRID: AB_2881315). FGD3 immunostaining was independently evaluated by two blinded investigators. The percentage of positive cells was scored as 0 (0%), 1 (1–25%), 2 (26–50%), 3 (51–75%), or 4 (76–100%). Staining intensity was scored as 0 (negative), 1 (light yellow), 2 (dark yellow), or 3 (brown). The final immunoreactive score (IRS) was calculated by summing the percentage and intensity scores (0–7). All IHC images were captured using a light microscope (Carl Zeiss, Oberkochen, Germany) at 10× and 40× magnification.

### 2.17. Plasmid Transfection, siRNA Knockdown, and FGD3 Overexpression

For FGD3 overexpression, H3122LR cells were transfected with an FGD3-overexpressing plasmid or an empty vector using Lipofectamine 3000 (Invitrogen, Carlsbad, CA, USA; cat# L3000015). For transient overexpression, cells were cultured for 24–48 h after transfection. For stable overexpression, cells were selected with puromycin (Sigma-Aldrich, St. Louis, MO, USA; cat# P8833). Transfection efficiency was confirmed by Western blot. The plasmid sequence is provided in Supplementary Table S2.

For FGD3 knockdown, parental ALK-positive H3122 and H2228 cells were transfected with siFGD3 or siControl using Lipofectamine 3000. The siRNA with the highest knockdown efficiency (>70%) was selected for each cell line (siRNA#3 for H3122 and siRNA#2 for H2228). Knockdown efficiency was confirmed by Western blot. The siRNA sequences are provided in Supplementary Table S3.

### 2.18. Colony Formation Assay

H3122LR cells transfected with an FGD3-overexpressing plasmid or an empty vector were seeded into 6-well plates (500 cells/well) and cultured for 10 days. Colonies were fixed with 4% paraformaldehyde (Biosharp, Hefei, China, Cat#BL539A), stained with crystal violet (Sigma-Aldrich, St. Louis, MO, USA, Cat#C0775), and counted. Colonies containing >50 cells were counted. Experiments were performed in triplicate and repeated at least three times independently.

### 2.19. CCK-8 Assay

Cell viability was assessed using the CCK-8 kti. Cells (2000/well) were seeded in 96-well plates and cultured for 1–4 days. CCK-8 solution was added (1:10 dilution), and plates were incubated for 1 h at 37 °C. Absorbance was measured at 450 nm. Each experiment was performed in triplicate and repeated at least three times independently.

### 2.20. Wound-Healing Assay

H3122LR cells transfected with an FGD3-overexpressing plasmid or an empty vector were seeded in 6-well plates and cultured to 90–100% confluence. A scratch was made using a 10 μL pipette tip, and serum-free medium was added. Wound closure was quantified using ImageJ. Experiments were performed in triplicate and repeated at least three times independently.

### 2.21. Cell Migration and Invasion Assays

For the transwell assay, 1 × 10^5^ cells were seeded in the upper chamber (8-μm pore size, Corning, Corning, NY, USA, Cat#3422) in serum-free medium, either uncoated (migration) or coated with Matrigel (BD Biosciences, Franklin Lakes, NJ, USA, Cat#356234). The lower chamber contained 600 µL of medium with 10% FBS. After 24 h (migration) or 48 h (invasion), cells on the lower surface of the membrane were fixed with 4% paraformaldehyde and stained with crystal violet. Cell numbers were quantified as the average number of cells per field of view in five randomly selected fields per well. Each experiment was performed in triplicate and repeated at least three times independently.

### 2.22. Statistical Analysis

All statistical analyses were performed using R (version 4.4.0; R Foundation for Statistical Computing, Vienna, Austria; RRID: SCR_001905) and GraphPad Prism (version 9.5; GraphPad Software, San Diego, CA, USA; RRID: SCR_002798). Data are presented as mean ± SD. Normality of data distribution was assessed using the Shapiro–Wilk test. For normally distributed variables, unpaired or paired Student’s *t*-tests and Pearson correlation were used. For non-normally distributed variables, the Wilcoxon rank-sum test (Mann–Whitney U test) and Spearman’s rank correlation were used. For categorical variables with small sample sizes, Fisher’s exact test was applied. ROC curves and AUC values were used to evaluate predictive performance. All tests were two-tailed, and *p* < 0.05 was considered statistically significant. Differential expression analysis, functional enrichment analysis, univariate and multivariate Cox regression, LASSO regression, and Kaplan–Meier survival analysis are described in the corresponding sections and are not repeated here.

## 3. Results

### 3.1. Acquisition of DE-ZNFs in LUAD and Functional Enrichment Analysis

**Figure 1:**
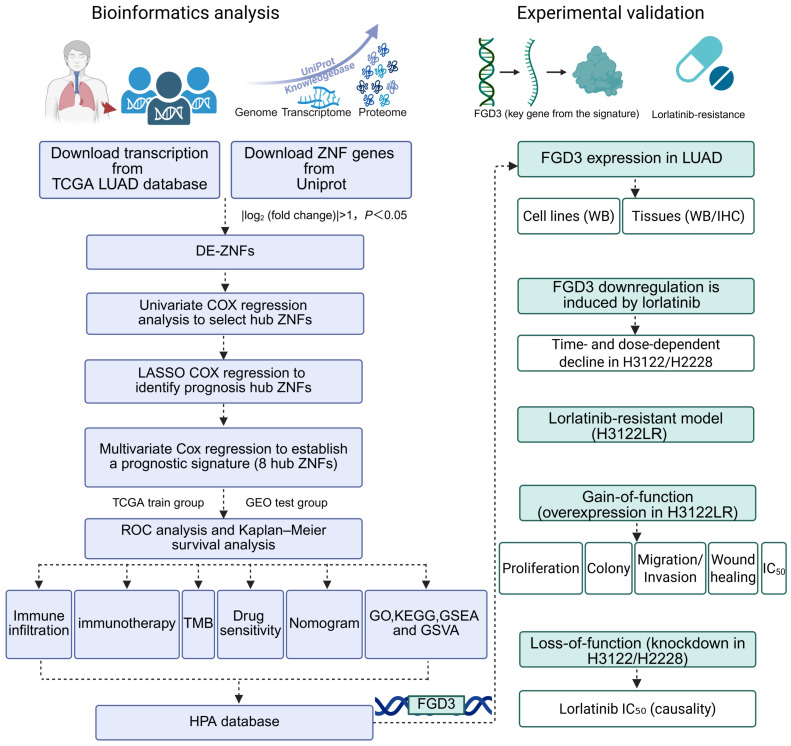
Flowchart showing the steps of this study. Blue: bioinformatics analysis; Green: experimental validation. Arrows indicate the sequential order.

**Figure 2:**
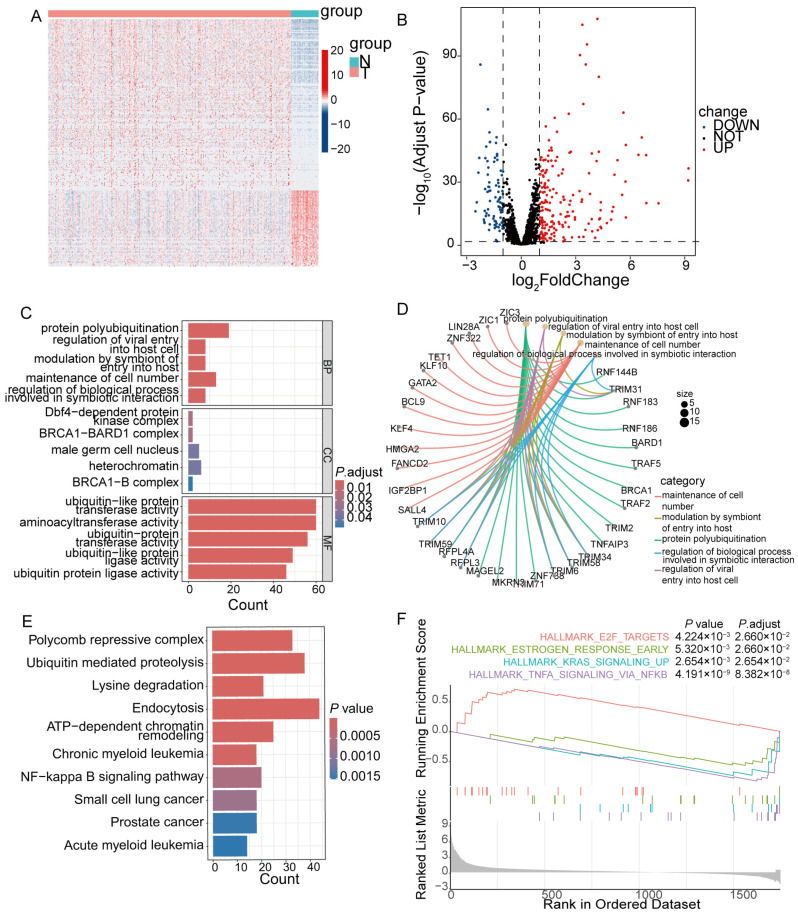
Differentially expressed ZNFs and functional analysis. (**A**,**B**) Heat map and volcano plot of ZNF related DEGs, showing 187 upregulated and 83 downregulated ZNF genes. Dashed lines indicate the thresholds for log_2_ fold change (±1). (**C**) Top 5 GO terms, as well as BP, CC, and MF enrichment results of DE-ZNFs. (**D**) The cnetplot showes the BP enrichment results. (**E**) Top 10 enriched pathways involving DE-ZNFs based on KEGG enrichment. (**F**) Pathways involving DE-ZNFs according to GSEA. The gray shadow indicates the distribution density of genes across the ranked list (x-axis: rank in ordered dataset; y-axis: running enrichment score).

The workflow of this study is illustrated in Figure 1. A total of 19,934 RNA-seq genes and clinical information for 513 LUAD patients and 58 normal samples were downloaded. Among them, 1834 ZNF genes were obtained. After differential expression analysis, 270 DE-ZNFs were identified, including 83 downregulated and 187 upregulated genes (Figure 2A,B). To evaluate the potential functions of DE-ZNFs in LUAD, GO enrichment analysis was performed. Sixteen biological process (BP) pathways, 5 cellular component (CC) pathways, and 23 molecular function (MF) pathways were identified, with the top five displayed. DE-ZNFs were mainly involved in the protein polyubiquitination process (Figure 2C,D). KEGG enrichment analysis revealed a total of 111 enriched pathways, including key biological processes such as ubiquitin-mediated proteolysis, lysine degradation, endocytosis, and chromatin remodeling (Figure 2E). Notable signaling pathways identified by GSEA and KEGG enrichment included TNFA signaling via NF-κB, KRAS signaling, and the E2F transcription factor pathway (Figure 2E,F), suggesting that DE-ZNFs function mainly in protein ubiquitination and are associated with inflammatory, oncogenic, and cell-cycle signaling pathways.

### 3.2. Development and Validation of the Prognostic Model for LUAD

**Figure 3:**
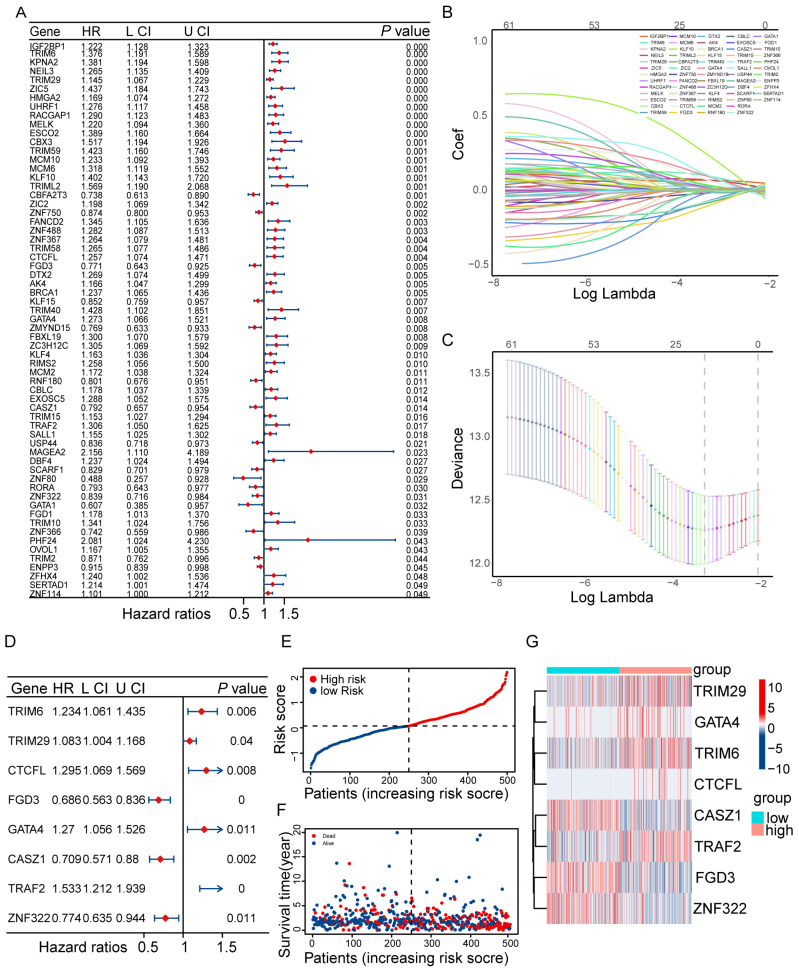
Development of the prognostic model based on OS-relevant DE-ZNFs. (**A**) Selection of 63 ZNF genes by univariate Cox regression analysis. Red diamonds represent hazard ratios (HRs); blue lines indicate 95% confidence intervals (CIs). (**B**,**C**) Nineteen ZNF genes underwent parameter selection via LASSO regression. Dashed vertical lines represent optimal λ values: λ.min (left, minimum cross-validation error) and λ.1se (right, largest λ within one SE of the minimum). (**D**) Multivariate Cox regression analysis-derived forest plot showing that 8 ZNF genes were the most prominent parameters to construct the best prognostic model. Red diamonds represent HRs; blue lines indicate 95% CIs; blue arrows denote CIs extending beyond the axis limits. (**E**) Distribution of the patients’ risk score. Vertical dashed line: cutoff between high- and low-risk groups; horizontal dashed line: median risk score. (**F**) Patients’ survival time with risk scores. Vertical dashed line: same risk group cutoff as in (**E**). (**G**) A heatplot showing expression of the 8 model genes in the high and low-risk groups.

**Figure 4:**
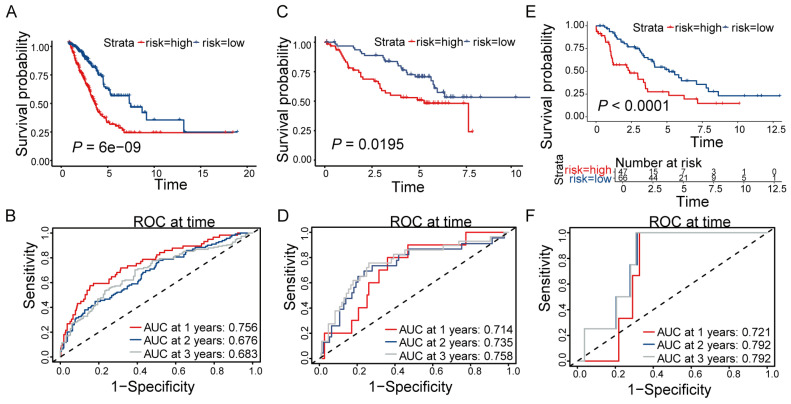
Validation of the prognostic model on internal and external LUAD cohorts. (**A**,**B**) OS survival curves and ROC curves for low- and high-risk subgroups in LUAD-TCGA cohort. (**C**–**F**) OS survival curves and ROC curves in GSE50081 and GSE26939 cohorts. The diagonal dashed line in ROC curves (**B,D,F**) represents the reference line (AUC = 0.5), indicating no discriminatory ability.

Univariate Cox regression analysis identified 63 DE-ZNFs significantly associated with OS in the TCGA training set (Figure 3A). After excluding samples with missing values or OS = 0, 500 cancer samples remained. LASSO regression was then applied to further select 19 DE-ZNFs (Figure 3B,C). Finally, multivariate Cox regression was used to establish the predictive model and identify prognostic-related genes: *TRIM6*, *TRIM29*, *CTCFL*, *FGD3*, *GATA4*, *CASZ1*, *TRAF2*, and *ZNF322* (Figure 3D). The risk score was calculated as follows: Risk score = (0.21 × ExpTRIM6) + (0.079 × ExpTRIM29) + (0.259 × ExpCTCFL) + (−0.377 × ExpFGD3) + (0.239 × ExpGATA4) + (−0.344 × ExpCASZ1) + (0.427 × ExpTRAF2) + (−0.256 × ExpZNF322). LUAD samples were then divided into high- and low-risk groups based on the median risk score. Risk curves and scatter plots illustrated that the high-risk group exhibited higher risk scores and mortality rates compared with the low-risk group (Figure 3E,F). The heatmap showed that *TRIM29*, *GATA4*, *TRIM6*, *CTCFL*, and *TRAF2* were highly expressed in the high-risk group, whereas *FGD3*, *CASZ1*, and *ZNF322* were highly expressed in the low-risk group (Figure 3G). Kaplan–Meier analysis demonstrated that patients in the high-risk group had significantly poorer OS than those in the low-risk group (Figure 4A; *p* < 0.001). Time-dependent ROC analysis yielded AUC values of 0.756 (95% CI: 0.712–0.800), 0.676 (95% CI: 0.622–0.730), and 0.683 (95% CI: 0.629–0.737) for 1-, 2-, and 3-year survival, respectively (Figure 4B). Two independent GEO validation datasets also confirmed shorter OS in the high-risk groups (Figure 4C; *p* = 0.0195 Figure 4E; *p* < 0.001). For the GSE50081 validation dataset, the 1-, 2-, and 3-year AUC values were 0.714 (95% CI: 0.620–0.808), 0.735 (95% CI: 0.652–0.818), and 0.758 (95% CI: 0.676–0.840), respectively; for GSE26939, the corresponding AUCs were 0.721 (95% CI: 0.622–0.820), 0.792 (95% CI: 0.707–0.877), and 0.792 (95% CI: 0.707–0.877) (Figure 4D,F). These results confirm the acceptable predictive performance of the prognostic model across independent cohorts.

### 3.3. The Prognostic Model Serves as a Dependable Indicator for Tumor Immunity

**Figure 5:**
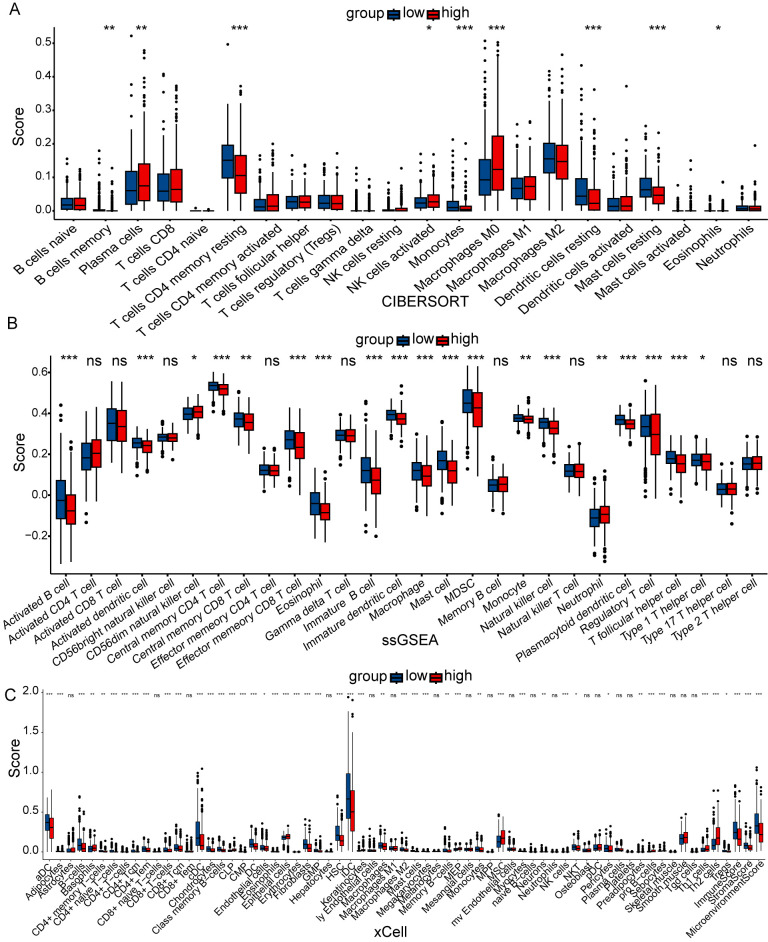
Tumor-immune microenvironment analysis of the high- and low-risk groups. (**A**) Relative abundance of 22 tumor-infiltrating immune cells estimated by CIBERSORT. Blue bars represent the low-risk group and red bars the high-risk group. (**B**) Relative infiltration levels of 28 immune cell subsets between the low- and high-risk groups in the TCGA-LUAD cohort. (**C**) xCell boxplot illustrating the relative abundance of infiltrating immune cell types in the high- and low-risk groups. Statistical significance: * *p* < 0.05, ** *p* < 0.01, *** *p* < 0.001; ns, not significant.

**Figure 6:**
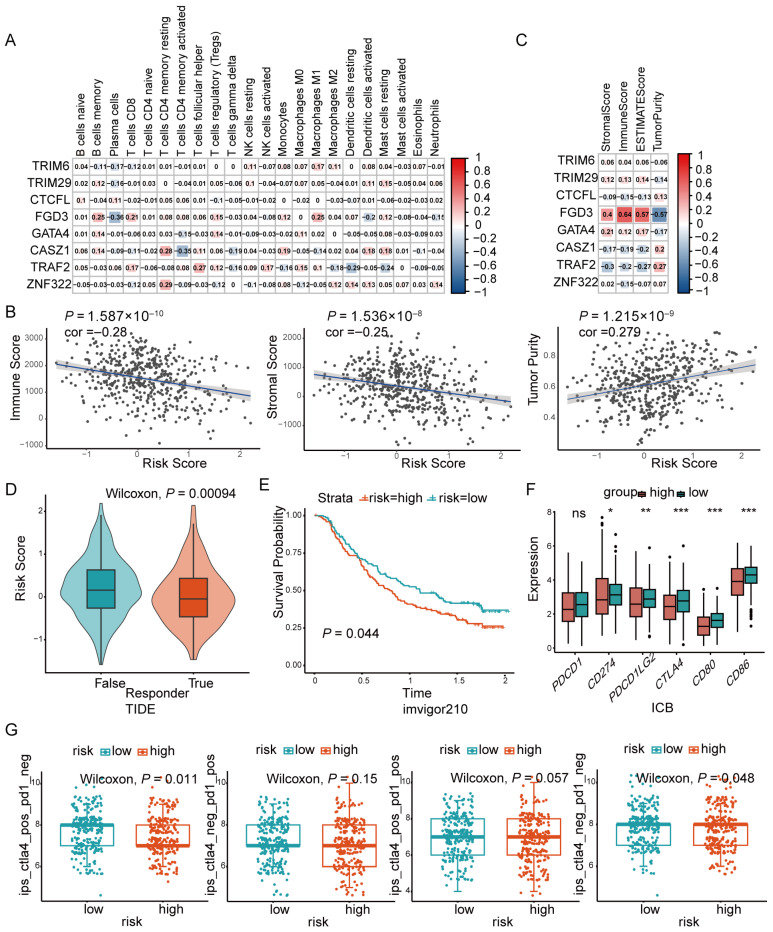
Predictive value of the ZNF signature-related prognostic model for immunotherapy. (**A**) Correlation between 22 tumor-infiltrating immune cells and the eight model genes. Colors represent Spearman correlation coefficients. (**B**) Correlation analysis between risk score and ESTIMATE scores (ImmuneScore, StromalScore, and ESTIMATE score) based on the ESTIMATE algorithm. The blue line represents the linear regression fit, and the gray shaded area indicates the 95% CI. (**C**) Correlation heatmap of ESTIMATE scores and the eight model genes. Colors represent Spearman correlation coefficients. (**D**) Predicted TIDE scores in the high- and low-risk groups (Wilcoxon test, *p* = 0.00094). (**E**) Exploratory analysis of predicted immunotherapy response in the high- and low-risk groups based on the IMvigor210 dataset. (**F**) Expression of ICB genes (PDCD1, CD274, PDCD1LG2, CTLA4, CD80, CD86) in the high- and low-risk groups (Wilcoxon test). (**G**) Comparison of IPSs between risk groups in LUAD. Statistical significance: * *p* < 0.05, ** *p* < 0.01, *** *p* < 0.001; ns, not significant.

According to the inferred results from CIBERSORT, ssGSEA, and xCell analyses, the low-risk group exhibited a higher proportion of immune cell infiltration (Figure 5), suggesting stronger tumor immunity compared with the high-risk LUAD patients. The prognostic-related gene *FGD3* showed positive correlations with the infiltration of memory B cells (ρ = 0.25, *p* < 0.001), CD8^+^ T cells (ρ = 0.21, *p* = 0.002), and M1 macrophages (ρ = 0.25, *p* < 0.001), while *ZNF322* and *CASZ1* were associated with increased resting CD4^+^ memory T cells (ρ = 0.28, *p* < 0.001; ρ = 0.29, *p* < 0.001) (Figure 6A). Patients in the high-risk group had lower immune scores (ρ = −0.28, *p* < 0.001), lower stromal scores (ρ = −0.25, *p* < 0.001), and higher tumor purity (ρ = 0.279, *p* < 0.001) based on the ESTIMATE algorithm (Figure 6B). The heatmap demonstrated that *FGD3* was significantly positively correlated with immune, stromal and ESTIMATE scores (ρ = 0.4, *p* < 0.001; ρ = 0.54, *p* < 0.001; ρ = 0.57, *p* < 0.001), but negatively correlated with tumor purity (ρ = −0.57, *p* < 0.001). In contrast, *CASZ1* and *TRAF2* exhibited opposite correlation patterns compared with *FGD3* (Figure 6C). The TIDE score predicted that the high-risk group exhibited poorer immunotherapy efficacy (*p* < 0.001) (Figure 6D). As an exploratory analysis, validation using the IMvigor210 dataset predicted that patients in the high-risk group had worse survival outcomes (Figure 6E; *p* = 0.044). To explore the relationship between risk score and immune checkpoint blockade (ICB) biomarkers, we found that expression levels of *PD-L1* (*p* = 0.012), *PD-L2* (*p* = 0.007), and *CTLA4* (*p* < 0.001) were lower in the high-risk group, suggesting a reduced response to therapies targeting these molecules (Figure 6F). *CD80* and *CD86*, key ligands for T-cell activation, were also downregulated in tumor cells from the high-risk group (*p* < 0.001 for all comparisons), which may contribute to immune escape (Figure 6F). Furthermore, based on the IPS, the low-risk group was predicted to derive greater benefit from anti CTLA4 therapy (Figure 6G). This comprehensive analysis elucidates the complex interactions between zinc finger proteins and immune therapy, confirming the prognostic model related to ZNFs as a valuable tool for guiding immunotherapy in LUAD.

### 3.4. Risk Score Performance in TMB, Enrichment and Drug Selection

**Figure 7:**
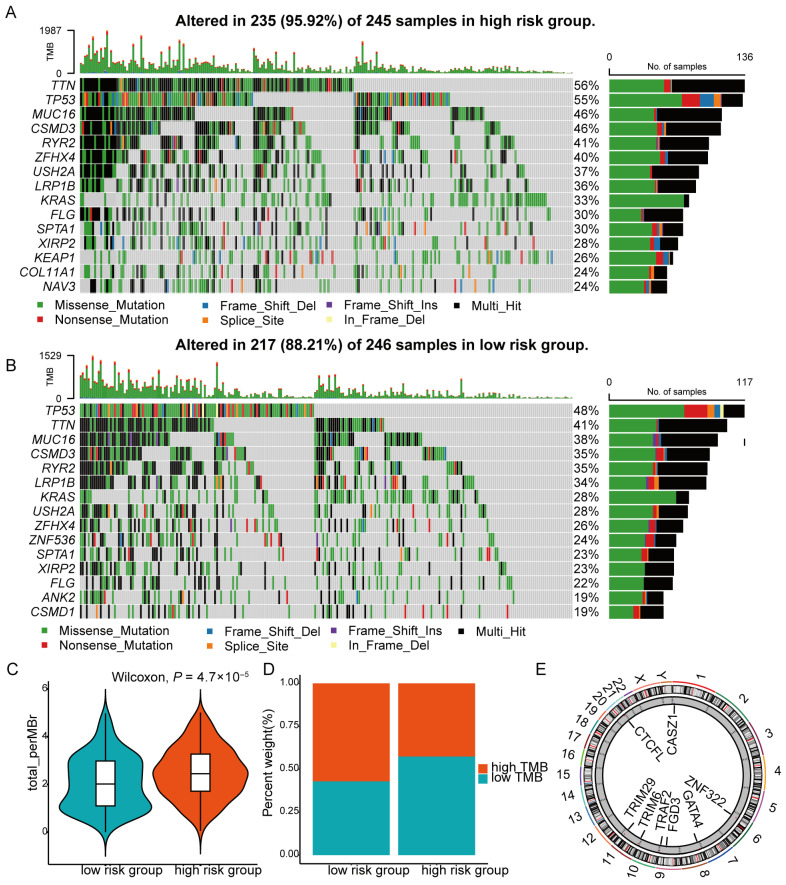
Mutation frequency in prognostic groups. (**A**) Oncoprint visualization of the mutation landscape in the high-risk group across LUAD samples from the TCGA cohort. (**B**) Oncoprint visualization of the mutation landscape in the low-risk group across LUAD samples from the TCGA cohort. (**C**,**D**) Violin plot (**C**) and proportional bar chart (**D**) showing that TMB was higher in the high-risk group. (**E**) Chromosomal locations of the eight prognostic model genes. Different colors on the outermost ring represent different chromosomes.

**Figure 8:**
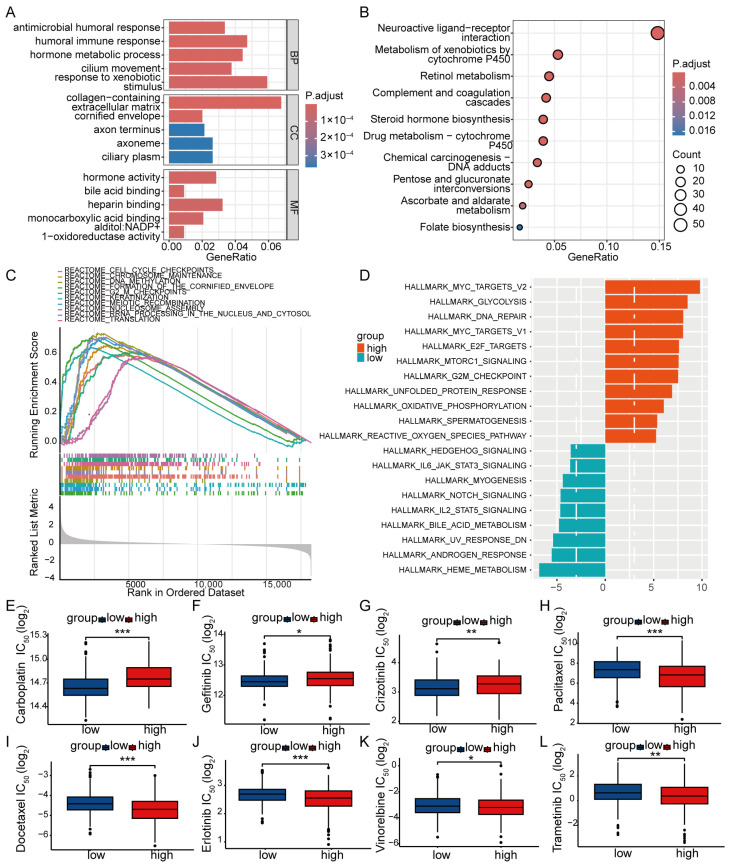
Functional enrichment and drug sensitivity prediction associated with the ZNF signature-related prognostic model in LUAD. (**A**) Top 5 GO terms (BP, CC, and MF) enriched in DE-ZNFs between the high- and low-risk groups. (**B**) Top 10 enriched KEGG pathways of DE-ZNF genes between the high- and low-risk groups. (**C**) GSEA pathways of DE-ZNF genes between the high- and low-risk groups. The gray shadow indicates the distribution density of genes across the ranked list (x-axis: rank in ordered dataset; y-axis: running enrichment score). (**D**) GSVA enrichment of DE-ZNF genes between the high- and low-risk groups. (**E**–**L**) Predicted drug sensitivity in the high- and low-risk groups. Patients in the high-risk group were predicted to be less sensitive to carboplatin (**E**), gefitinib (**F**), and crizotinib (**G**), whereas low-risk patients were predicted to be more sensitive to paclitaxel (**H**), docetaxel (**I**), erlotinib (**J**), vinorelbine (**K**), and trametinib (**L**) (Wilcoxon test). Predicted IC_50_ values are shown on a log_2_ scale; lower values indicate higher predicted sensitivity. Statistical significance: * *p* < 0.05, ** *p* < 0.01, *** *p* < 0.001.

To investigate the molecular consequences of genetic differences between the high- and low-risk groups and to better understand the biological processes associated with poor survival in the high-risk group, we analyzed the genomic variability of the ZNF model within the TCGA LUAD cohort. Oncoprint visualization revealed that somatic mutation frequency was higher in high-risk patients (95.92%) compared with the low-risk group (88.21%). The top 15 mutated genes were identified, with missense mutations and multi-hit mutations being the most common types. Notably, mutation rates of *TP53* (55% vs. 48%), *TTN* (56% vs. 41%), *MUC16* (46% vs. 38%), and *KRAS* (33% vs. 28%) were significantly elevated in the high-risk group relative to the low-risk group (Figure 7A,B). Consistently, TMB was significantly higher in the high-risk group (*p* < 0.001) (Figure 7C,D). However, stratification of patients based on the median TMB showed that those with higher TMB had longer OS (*p* = 0.025) (Figure S1). The chromosomal locations of the eight prognosis-related ZNF genes are displayed (Figure 7E). GO functional enrichment analysis of DE-ZNFs between risk groups indicated predominant localization in the collagen-containing extracellular matrix, cornified envelope, and axon terminus. Molecular functions enriched in these genes included hormone activity, bile acid binding, and heparin binding (Figure 8A). After ID conversion, 311 downregulated and 554 upregulated genes were retained for further analysis. KEGG pathway analysis revealed significant differences between risk groups in pathways such as chemical carcinogenesis–DNA adducts, neuroactive ligand–receptor interaction, steroid hormone biosynthesis, and drug metabolism via cytochrome P450 (Figure 8B). GSEA identified 229 significantly enriched pathways (FDR < 0.25), with the top 10 pathways predominantly enriched in the high-risk group. These pathways were associated with cell division, cell cycle regulation, DNA replication, transcription and translation, chromosome maintenance, DNA methylation, and keratinization (Figure 8C), potentially explaining the shorter OS observed in the high-risk group. GSVA enrichment analysis further demonstrated that poor prognosis in the high-risk group was associated with activation of the MYC signaling pathway, glycolysis, mTOR signaling, DNA repair, E2F transcription factors, unfolded protein response, and oxidative phosphorylation (Figure 8D). These findings suggest potential therapeutic targets for LUAD patients involving ZNFs. To identify potential chemotherapeutic and targeted agents for high-risk LUAD patients, we performed drug sensitivity prediction using available pharmacogenomic datasets. The high-risk group exhibited potential resistance to carboplatin (*p* < 0.001), gefitinib (*p* = 0.037), and crizotinib (*p* = 0.006), whereas increased sensitivity was predicted for paclitaxel (*p* < 0.001), docetaxel (*p* < 0.001), erlotinib (*p* < 0.001), vinorelbine (*p* = 0.039), and trametinib (*p* = 0.009) (Figure 8E–L). These findings suggest that the risk score may guide personalized drug selection for LUAD patients.

### 3.5. Nomogram Performance and Validation

**Figure 9:**
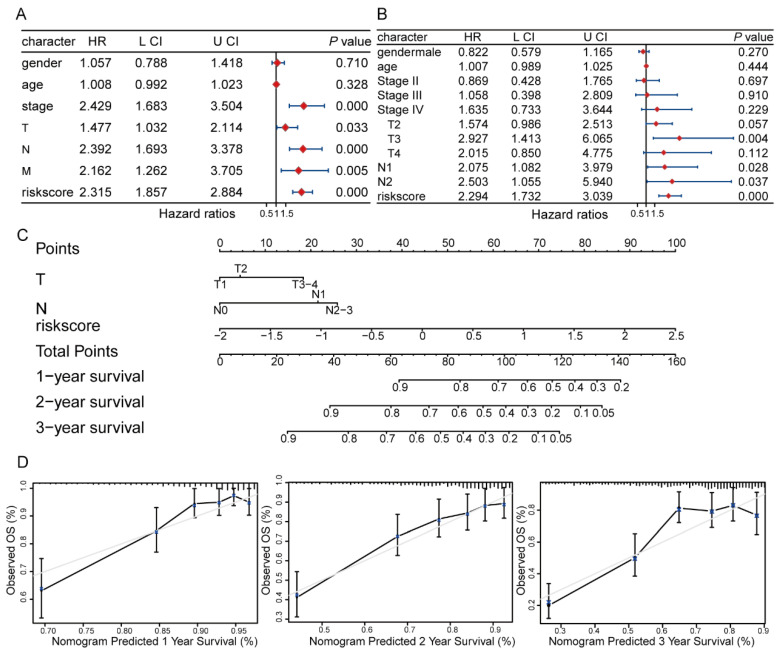
Evaluation of the prognostic signature for predicting OS in LUAD patients. (**A**) Univariate Cox regression analysis of stage, age, gender, grade, race, T stage, N stage, M stage, and risk score. Red diamonds represent HRs; blue lines indicate 95% CIs. (**B**) Multivariate Cox regression analysis of stage, age, gender, grade, race, T stage, N stage, M stage, and risk score. Red diamonds represent HRs; blue lines indicate 95% CIs. (**C**) Nomogram based on T stage, N stage, and risk score. (**D**) Calibration curves of the nomogram and observed 1-, 2-, and 3-year survival of LUAD patients. The gray line represents ideal performance, and the black line represents the actual performance of the signature.

Clinical information for the high- and low-risk groups was downloaded, and inter-group comparisons were performed using Fisher’s exact test, as summarized in Table 1. The high-risk group exhibited a higher proportion of male patients (*p* < 0.001), advanced T stage (*p* < 0.001), increased lymph node metastasis (*p* = 0.007), and higher overall stage (*p* < 0.001). Univariate and multivariate Cox regression analyses were conducted on clinicopathological factors—including gender, age, stage, TNM classification, and risk score—to identify independent prognostic factors for LUAD. Variables with *p* < 0.05 in both analyses were deemed independent risk factors; accordingly, T stage (HR = 1.48, 95% CI: 1.03–2.11, *p* = 0.033), N stage (HR = 2.39, 95% CI: 1.69–3.38, *p* < 0.001), and risk score (HR = 2.30, 95% CI: 1.85–2.88, *p* < 0.001) were selected (Figure 9A,B). These independent prognostic factors were then integrated to construct a nomogram, facilitating the prediction of 1-, 2-, and 3-year OS probabilities in the TCGA-LUAD cohort. Due to the limited sample size in the T4 and N3 categories, singular fit issues arose during Cox regression modeling; hence, T3 and T4 were combined, as were the N2 and N3 groups (Figure 9C). Calibration plots for 1-, 2-, and 3-year OS demonstrated good agreement between predicted and observed survival outcomes, indicating that the nomogram has satisfactory predictive accuracy (Figure 9D).

### 3.6. Expression and Survival Analysis of Eight Prognostic Genes

**Figure 10:**
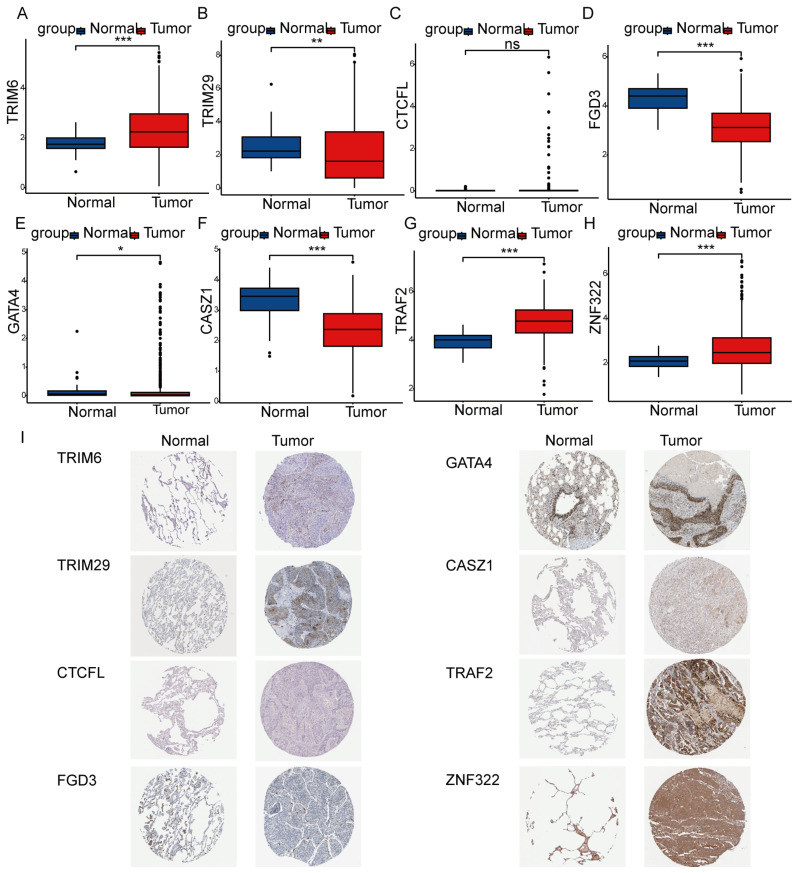
Expression validation of the eight risk model genes in the TCGA and HPA databases. (**A**–**H**) Expression levels of TRIM6, TRIM29, CTCFL, FGD3, GATA4, CASZ1, TRAF2, and ZNF322 in LUAD vs. normal tissues from the TCGA dataset. All statistical tests were performed using the Wilcoxon test. Statistical significance: * *p* < 0.05, ** *p* < 0.01, *** *p* < 0.001; ns, not significant. (**I**) Protein expression patterns of the eight risk model genes in LUAD and normal tissues based on IHC data from the HPA database.

We validated the differential expression of the eight ZNF prognostic model genes between LUAD patients and normal samples in the TCGA-LUAD dataset. The results showed that *TRIM6* (*p* < 0.001), *GATA4* (*p* = 0.041), *TRAF2* (*p* < 0.001), and *ZNF322* (*p* < 0.001) were significantly upregulated in LUAD patients, whereas *TRIM29* (*p* = 0.008), *FGD3* (*p* < 0.001), and *CASZ1* (*p* < 0.001) were downregulated (Figure 10A–H). Protein expression levels of these eight prognostic genes in LUAD and normal tissues were further analyzed using data from the Human Protein Atlas (HPA) database. The protein expression patterns of GATA4, TRAF2, and ZNF322 were consistent with their RNA expression trends, showing elevated levels in LUAD patients. Similarly, FGD3 and CASZ1 exhibited lower protein expression in patients, consistent with RNA data. No significant differences were observed between tumor and normal tissues for CTCFL at either the protein or RNA level. Notably, TRIM6 protein was expressed at low levels in both tumor and normal tissues, and TRIM29 protein expression showed an inverse pattern relative to its RNA expression (Figure 10I). Kaplan–Meier survival analysis demonstrated that higher expression of *TRIM6* (*p* < 0.001), *TRIM29* (*p* = 0.014), and *TRAF2* (*p* = 0.024), along with lower expression of *FGD3* (*p* < 0.001), *CASZ1* (*p* = 0.007), and *ZNF322* (*p* = 0.012), were associated with poorer OS in LUAD patients. Expression levels of the remaining two genes did not show significant prognostic relevance (Figure S2).

### 3.7. Loss of FGD3 Contributes to LUAD Cell Proliferation and Drug Resistance

**Figure 11:**
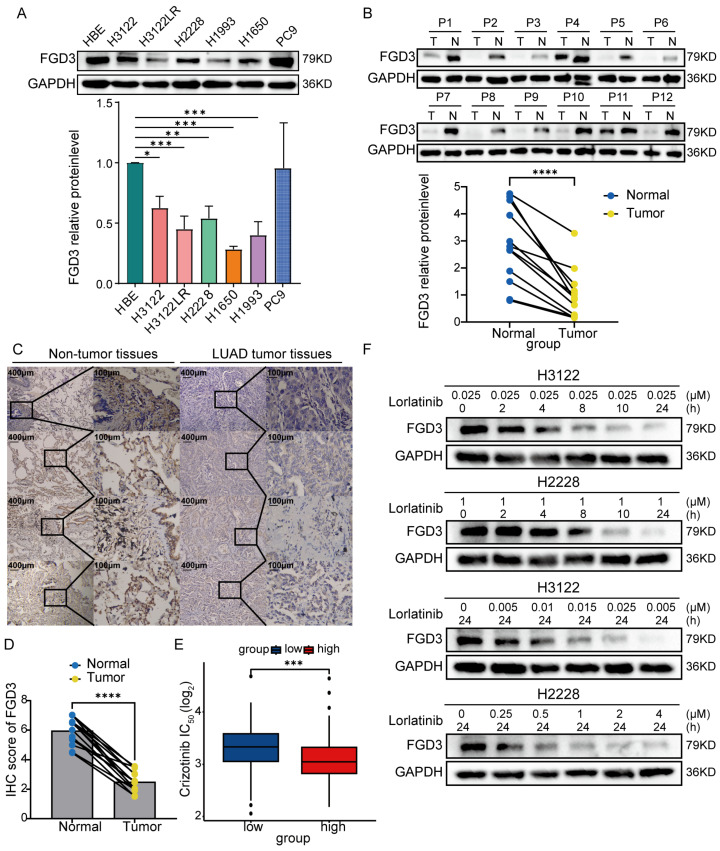
FGD3 is downregulated in LUAD and possibly involved in drug sensitivity. (**A**) Western blot analysis of FGD3 protein expression in LUAD cell lines compared with normal bronchial epithelial (HBE) cells. Quantification data are presented as mean ± SD from three independent experiments (unpaired *t*-test). (**B**) FGD3 protein levels in 12 paired LUAD tumor tissues (T) and adjacent normal tissues (N) (paired *t*-test). (**C**) Representative IHC staining of FGD3 in LUAD and adjacent normal tissues (images from 4 representative cases). (**D**) Quantitative analysis of FGD3 IRS in LUAD tissues versus adjacent normal tissues (*n* = 14) (paired *t*-test). (**E**) Predicted drug sensitivity showed that the low-FGD3-expression group was less sensitive to the first-generation ALK inhibitor crizotinib (Wilcoxon test). (**F**) Time- and concentration-dependent downregulation of FGD3 protein in ALK-positive H3122 and H2228 cells following lorlatinib treatment. Statistical significance: * *p* < 0.05, ** *p* < 0.01, *** *p* < 0.001, **** *p* < 0.0001.

Among the eight prognostic-related genes, the FYVE zinc finger domain-containing FGD3 gene exhibited the strongest correlation with ESTIMATE scores and a relatively high correlation coefficient within the prognostic model. Consequently, we prioritized this ZNF-associated gene for downstream validation. Paired RNA-seq data from TCGA showed significantly lower *FGD3* expression in LUAD tissues compared with normal samples (*p* < 0.001) (Figure S3A), and consistent results were observed in two paired GEO datasets (GSE32863: *p* < 0.001; GSE75037: *p* < 0.001) (Figure S3B,C). Protein expression of FGD3 was further validated in LUAD cell lines and normal bronchial epithelial (HBE) cells. Except for PC9, FGD3 protein levels in all cells (H3122, H2228, H1650, and H1993) were significantly lower than those in HBE cells (Figure 11A). The uncropped blots for Figure 11A are shown in Supplementary Original Blot Figure. A similar expression pattern was confirmed in 12 paired LUAD tumor and adjacent normal tissues, with FGD3 expression significantly lower in tumors (95%CI: −2.47 to −1.19, *p* < 0.0001) (Figure 11B). The uncropped blots for Figure 11B are shown in Supplementary Original Blot Figure. The IHC results showed that compared with LUAD tissues, FGD3 expression was significantly higher in normal tissues (*p* < 0.001). Cellular localization revealed that FGD3 was expressed in both the cytoplasm and nucleus, with greater expression in the cytoplasm. FGD3 was more highly expressed in the epithelial cells of the trachea and bronchi (Figure 11C,D). GO, KEGG, and GSEA enrichment analyses revealed that genes differentially expressed with *FGD3* were mainly enriched in cilium movement and cell adhesion pathways in LUAD (Figure S3D–F). Drug sensitivity prediction estimated that the low-*FGD3*-expression group was less sensitive to the first-generation ALK inhibitor crizotinib (*p* < 0.001) (Figure 11E). To further investigate whether FGD3 expression is affected by ALK inhibitor treatment, we examined lorlatinib-resistant H3122 cells (H3122LR) (Figure S4) and found that FGD3 expression was significantly decreased compared with parental cells (*p* = 0.039) (Figure 11A). Consistently, FGD3 expression showed a dose- and time-dependent decline in ALK-positive H3122 and H2228 cells following prolonged lorlatinib treatment and increasing drug concentration (Figure 11F). The uncropped blots for Figure 11F are shown in Supplementary Original Blot Figure. Together, these results suggest that FGD3 downregulation is associated with reduced sensitivity to ALK inhibitors and may be induced by prolonged drug exposure.

To further establish that FGD3 downregulation causally contributes to lorlatinib resistance, we knocked down FGD3 in ALK-positive H3122 and H2228 cells using specific siRNAs (sequences provided in Table S3). Western blotting confirmed knockdown efficiencies exceeding 70% (siRNA#3 for H3122 and siRNA#2 for H2228; Figure S5A,B,D,E). The uncropped blots for Supplementary Figure S5A,D are shown in Supplementary Original Blot Figure. Compared with the siNC control, FGD3 knockdown significantly increased the IC_50_ of lorlatinib in both cell lines (H3122: *p* < 0.01; H2228: *p* < 0.05; Figure S5C,F), demonstrating that loss of FGD3 reduces lorlatinib sensitivity.

**Figure 12:**
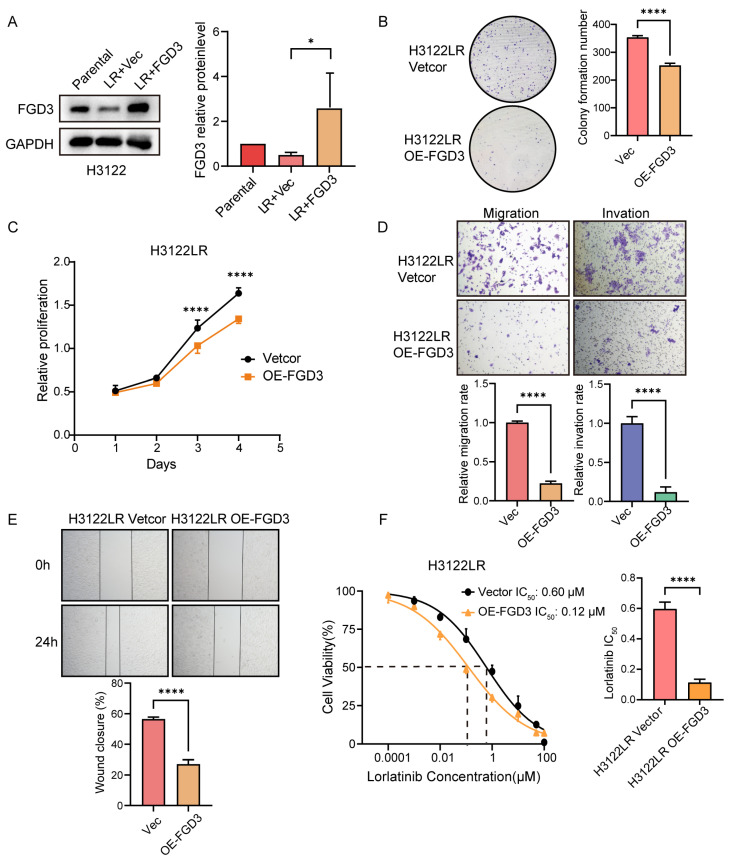
Functional validation of FGD3 in H3122LR cells. (**A**) FGD3 protein expression in H3122LR cells after transfection with empty vector (Vec) or FGD3-overexpressing plasmid (OE-FGD3). Quantitative data are presented as mean ± SD from three independent experiments (unpaired *t*-test). (**B**) Colony formation assay of H3122LR cells after transfection. (**C**) Cell viability of H3122LR cells after transfection. (**D**) Migration and invasion of H3122LR cells after transfection. (**E**) Wound-healing assay of H3122LR cells after transfection. (**F**) IC_50_ curves of lorlatinib in H3122LR cells after transfection. For (**B**–**F**), quantitative data are presented as mean ± SD from three independent experiments (unpaired *t*-test). Statistical significance: * *p* < 0.05, **** *p* < 0.0001.

To determine whether restoring FGD3 expression could reverse these effects, the FGD3 plasmid was transfected into H3122LR cells, and transfection efficiency was confirmed by Western blot (Figure 12A). The uncropped blot for Figure 12A is shown in Supplementary Original Blot Figure. Colony formation assays showed that *FGD3* overexpression markedly reduced the number of colonies formed by H3122LR cells (*p* < 0.001) (Figure 12B). Consistently, cell viability was significantly decreased upon *FGD3* overexpression (*p* < 0.001) (Figure 12C). Furthermore, Transwell assays demonstrated that upregulation of *FGD3* substantially impaired both migration and invasion capabilities (*p* < 0.001 for both) (Figure 12D). Wound-healing assays further indicated that *FGD3* overexpression attenuated the cells’ wound closure ability (*p* < 0.001) (Figure 12E). Finally, *FGD3* overexpression partially reversed lorlatinib resistance in H3122LR cells, as evidenced by a significant reduction in IC_50_ (Figure 12F).

## 4. Discussion

ZNFs constitute the largest family of transcription factors in mammalian cells and are involved in tumor initiation, progression, metastasis, and drug resistance by regulating transcriptional and translational processes [31], highlighting their potential as novel therapeutic targets. ZNFs have been described as a double-edged sword, exhibiting both oncogenic and tumor-suppressive functions, and serving as reliable prognostic biomarkers [32]. Numerous clinicopathological datasets, prognostic data, and ZNF-based cancer prognostic models have been developed for various cancers, including pancreatic cancer, soft tissue sarcoma, colon adenocarcinoma, esophageal cancer, breast cancer, and osteosarcoma [33,34,35,36,37,38,39]. Many prognostic models are currently utilized in clinical practice; for example, the Sokal, Euro, EUTOS, and ELTS scoring systems are employed to evaluate prognosis in chronic myeloid leukemia (CML) [40]. However, despite LUAD being the most prevalent malignant tumor in China, no prognostic model based on zinc finger proteins has yet been established for this disease. Our study fills this gap by developing the first ZNF-based prognostic signature for LUAD.

In this study, we identified 270 DE-ZNFs between LUAD and normal tissues. Through univariate Cox, LASSO, and multivariate Cox regression analyses, we developed an eight-gene prognostic signature (*TRIM6*, *TRIM29*, *CTCFL*, *FGD3*, *GATA4*, *CASZ1*, *TRAF2*, *ZNF322*) that effectively predicts OS. This model was rigorously validated in two independent GEO cohorts using Kaplan–Meier survival curves and time-dependent ROC analyses. Subsequently, we extended our investigation to assess immunotherapy responses between the high- and low-risk groups. Immune infiltration analysis revealed that the high-risk group exhibited an immune “desert” phenotype, characterized by relatively low levels of activated B cells and natural killer cells, as well as dendritic cells, which are key players in antigen presentation and immune activation [41]. Furthermore, CD4^+^ memory T cells, which play a critical role in tumor immune responses by maintaining long-term immune memory and enhancing anti-tumor activity [42], were diminished in the high-risk group. The deficiency of CD4+ memory T cells may reflect tumor immune escape mechanisms that suppress immune surveillance. Consistently, ESTIMATE analysis, TIDE scores, and IMvigor210 data predicted that the high-risk group had lower immune scores, higher tumor purity, and poorer responses to immunotherapy. Moreover, expression levels of PD-L1, PD-L2, and CTLA-4 were reduced in the high-risk group, potentially impairing T-cell recognition and indicating a poor response to immune checkpoint inhibitors [43,44,45]. IPS predictions aligned with these findings, suggesting that the high-risk group may be less responsive to anti-CTLA-4 therapy. Additionally, CD80 and CD86, essential co-stimulatory molecules on dendritic cells and B cells that facilitate T-cell activation [46], were expressed at lower levels in the high-risk group. This suggests impaired antigen-presenting cell function and insufficient immune activation, which may contribute to immune escape.

To better understand the molecular characteristics between different groups, TMB analysis was carried out. The progressive accumulation of somatic mutations throughout life may contribute to carcinogenesis [47]. The overall mutation rate was higher in the high-risk group (95.92% vs. 88.21%), suggesting greater tumor heterogeneity. TP53, a classic tumor suppressor which is crucial for a variety of cellular processes, including DNA repair, cell cycle arrest, and apoptosis [48], is the most commonly mutated in human cancers, including NSCLC. Co-mutations of TP53 and other driver genes further contribute to the poor prognosis of lung cancer [49]. In addition, KRAS is a proto-oncogene, and KRAS mutations are particularly prevalent in 30% of LUAD [50]. TTN is also frequently mutated in a variety of cancers, overexpression of TTN-AS1 correlates with poor prognosis in lung cancer [51]. The MUC16 mutation is reported to decrease the immune response in LUAD [52]. Our results showed higher mutation frequencies of TP53 (55% vs. 48%), TTN (56% vs. 41%), KRAS (33% vs. 28%), and MUC16 (46% vs. 38%) in the high-risk group compared to the low-risk group, which may partly explain the poorer prognosis of the high-risk patients. Previous studies reported higher TMB results in more neo-antigens, increasing chances for T-cell recognition, and clinically correlates with better immune checkpoint inhibitor outcomes [28]. In line with this, our results confirmed that when patients were stratified solely by TMB level (regardless of risk group), those with higher TMB had longer OS. However, when stratified by the risk score, the high-risk group showed higher TMB but exhibited a poorer response to immunotherapy. This apparent paradox can be resolved by examining the immune microenvironment of the high-risk group. The high-risk group displayed an immune-desert phenotype, characterized by reduced infiltration of multiple immune cell types, including dendritic cells, activated B cells, NK cells, and effector T-cell subsets, indicating defective antigen presentation and a lack of effector cells. In addition, expression levels of immune checkpoints (PD-L1, PD-L2, and CTLA-4) and co-stimulatory molecules (CD80/CD86) were significantly decreased in the high-risk group. Therefore, even though the high-risk group carries more mutations (higher TMB), the absence of a competent immune microenvironment fails to support effective T-cell priming, thereby blunting the potential benefit of high TMB. This explains why high TMB in the high-risk group does not translate into better immunotherapy response. TMB alone is therefore an imperfect biomarker for immunotherapy response; integrating TMB with immune contexture is necessary for accurate prediction. This aligns with the literature suggesting that composite predictors incorporating factors such as MHC and T-cell receptor repertoire are needed [6].

We observed that DEGs between risk groups were enriched in pathways related to cellular metabolism, proliferation, genomic stability, and gene expression regulation. These aberrant molecular processes likely cooperate to accelerate tumorigenesis and progression in the high-risk group. GSVA revealed enrichment of MYC and mTOR signaling pathways in high-risk LUAD, suggesting their potential as therapeutic targets. Furthermore, enrichment of DNA damage repair, E2F transcription factor, and unfolded protein response pathways indicated that tumor cells in the high-risk group may exhibit enhanced DNA repair capacity, sustained proliferative signaling [53] and disrupted endoplasmic reticulum proteostasis [54], thereby contributing to increased treatment resistance and tumor survival. Additionally, the risk assessment model could serve as a reliable tool to guide clinical treatment selection. For instance, our in silico predictions suggest that patients in the high-risk group may be less responsive to carboplatin, gefitinib, and crizotinib, while showing predicted higher sensitivity to paclitaxel, docetaxel, erlotinib, vinorelbine, and trametinib. These findings require prospective experimental and clinical validation. Moreover, we found that the risk score significantly correlated with clinicopathological parameters such as T stage and N stage, which supported the development of a predictive nomogram with high accuracy for OS.

Of the eight genes comprising our prognostic signature, TRIM6, GATA4, TRAF2, and ZNF322 were highly expressed in LUAD samples, whereas TRIM29, FGD3, and CASZ1 showed lower expression. Similar protein-level expression patterns were confirmed using the HPA database. Kaplan–Meier survival analysis suggested that TRAF2, TRIM29, and TRIM6 may promote carcinogenesis in LUAD, while CASZ1, FGD3, and ZNF322 likely act as tumor suppressors. However, TRIM29 showed a discordant pattern: although its mRNA was downregulated in tumor tissues, HPA data revealed relatively higher protein levels in tumors. This mRNA–protein discordance suggests post-transcriptional regulation, possibly through altered protein turnover or post-translational modifications. Notably, Kumari et al. reported that TRIM29 positivity in LUAD is only 30.8%, indicating heterogeneous expression [55]. Given the limited sample size of the HPA database, this discrepancy warrants direct validation in future studies using matched LUAD clinical samples. As reported in the previous literature, TRAF2 is an oncogenic protein involved in TNF-mediated apoptosis in NSCLC via NFκB [56]. Accumulating evidence suggests that TRIM29 is also a transcription factor involved in NSCLC carcinogenesis via binding RAD50 and confirmed to be highly expressed in anlotinib-resistant NSCLC cells [57]. Previous studies have proposed that TRIM6 acts as an E3-ubiquitin ligase and reduces ferroptosis and chemosensitivity in lung cancer [58]. Studies employing in vitro overexpression/knockdown, animal models, and IHC have defined ZNF322 as an oncogenic transcription factor in lung cancer [59,60,61,62]. Our data, in contrast, show the opposite. Notably, an independent bioinformatics study on LUAD also identified ZNF322 as a protective factor [63], consistent with our findings. We propose that this discrepancy may arise from methodological differences: cell line models cannot fully recapitulate the complex tumor microenvironment, whereas patient-derived transcriptomic data may reflect the net effect of ZNF322 in a clinical context. Thus, our findings do not negate previous studies but rather reveal a distinct role for ZNF322 in LUAD at the patient level. The role of CASZ1 varies across different types of tumors, and its role in lung cancer is also controversial. According to TCGA data, CASZ1 is expressed at low levels in LUAD samples. However, a study has reported that CASZ1 promotes migration, invasion, and metastasis in lung cancer [64]. GATA4 has been shown to function as an essential tumor suppressor in lung cancer both in vitro and in vivo [65].

FGD3 regulates actin cytoskeleton dynamics and cell morphology and suppresses cancer cell migration, correlating with favorable prognosis in breast and pancreatic cancers [66,67,68,69,70]. Here, we demonstrate for the first time that FGD3 protein expression is markedly reduced in LUAD tissues and cell lines and is further decreased in lorlatinib-resistant cells. Consistently, FGD3 knockdown in parental ALK-positive H3122 and H2228 cells significantly increased lorlatinib IC_50_, further supporting that loss of FGD3 contributes to drug resistance. Functional assays showed that FGD3 overexpression partially restores lorlatinib sensitivity in H3122LR cells, indicating that loss of FGD3 contributes to drug resistance. GO and KEGG analyses linked FGD3 to cilium movement and cell adhesion-related pathways, consistent with the increased membrane protrusions and cytoskeletal remodeling observed during resistance acquisition. Together with previous reports showing that FGD3 promotes actin reorganization and filopodia formation, these findings support a role for FGD3 in regulating cytoskeletal plasticity in LUAD [69]. Notably, recent studies have identified FGD3 as a key mediator of chemotherapy-induced plasma membrane rupture and immunogenic lytic cell death via the Cdc42–ARP2/3 axis [71]. In this context, further downregulation of FGD3 in lorlatinib-resistant cells may represent an adaptive mechanism to evade stress-induced cell death. Thus, FGD3 suppression may simultaneously enhance structural plasticity and promote survival under sustained drug pressure. Collectively, our results suggest that FGD3 acts as a context-dependent regulator of cytoskeletal remodeling and cell death execution. Its downregulation facilitates lorlatinib resistance, whereas restoring FGD3 expression partially reverses this phenotype, highlighting its potential as a biomarker of therapeutic response and a candidate target for overcoming ALK inhibitor resistance in LUAD.

Strengths and limitations of this study should be considered. The systematic identification of DE-ZNFs, rigorous model validation across multiple cohorts, and functional evidence linking FGD3 to lorlatinib resistance are key strengths. However, the prognostic model was built solely on public datasets and awaits prospective validation. Additionally, the IMvigor210 dataset used to predict immunotherapy response is derived from urothelial carcinoma patients, not LUAD patients. Given the substantial differences in tumor immune microenvironment and mutation profiles between these cancer types, this cross-cancer analysis should be considered exploratory. Moreover, although we propose FGD3 as a candidate biomarker for lorlatinib resistance, this finding requires prospective validation in independent clinical cohorts, particularly those comprising LUAD patients receiving lorlatinib or other ALK inhibitor therapy. FGD3 functional experiments were limited to in vitro assays without in vivo confirmation, and immune infiltration analysis relied on transcriptomic deconvolution rather than direct measurements. Moreover, we focused solely on ZNF family genes; other DE gene sets (e.g., non-ZNF genes) were not evaluated and warrant future investigation. In addition, functional validation of the other seven prognostic genes (TRIM6, TRIM29, CTCFL, GATA4, CASZ1, TRAF2, ZNF322) was not performed in this study and remains to be investigated in future work. Future studies should prospectively validate the signature, investigate the mechanistic basis of FGD3-mediated resistance, and evaluate its therapeutic potential in preclinical models.

## 5. Conclusions

This study establishes a promising eight-gene prognostic signature based on ZNFs for LUAD, providing a potential tool for risk stratification and individualized therapeutic decision making. Importantly, we identify FGD3 as a bronchial epithelial enriched gene whose downregulation correlates with poor prognosis and lorlatinib resistance; restoring FGD3 expression partially reverses resistance, highlighting its promise as a candidate biomarker and therapeutic target in LUAD that warrants further prospective validation in ALK inhibitor-treated patient cohorts.

## Figures and Tables

**Table 1:** Baseline clinical information table for high- and low-risk groups.

	Overall (*n* = 500)	High (*n* = 250)	Low (*n* = 250)	*p*
Gender (%)				
Female	271 (54.2)	114 (45.6)	157 (62.8)	<0.001
Male	229 (45.8)	136 (54.4)	93 (37.2)	
Age (mean)				0.016
	65.24 (10.07)	64.14 (10.87)	66.33 (9.10)	
T (%)				<0.001
T1	167 (33.6)	65 (26.2)	102 (41.0)	
T2	269 (54.1)	135 (54.4)	134 (53.8)	
T3	43 (8.7)	34 (13.7)	9 (3.6)	
T4	18 (3.6)	14 (5.6)	4 (1.6)	
N (%)				0.007
N0	323 (66.1)	147 (59.3)	176 (73.0)	
N1	95 (19.4)	55 (22.2)	40 (16.6)	
N2	69 (14.1)	44 (17.7)	25 (10.4)	
N3	2 (0.4)	2 (0.8)	0 (0.0)	
M (%)				0.375
M0	334 (93.3)	170 (91.9)	164 (94.8)	
M1	24 (6.7)	15 (8.1)	9 (5.2)	
Stage (%)				<0.001
Stage I	269 (54.6)	107 (43.5)	162 (65.6)	
Stage II	119 (24.1)	69 (28.0)	50 (20.2)	
Stage III	80 (16.2)	54 (22.0)	26 (10.5)	
Stage IV	25 (5.1)	16 (6.5)	9 (3.6)	

## Data Availability

The datasets analyzed in this study are publicly available from TCGA (https://portal.gdc.cancer.gov/) and GEO (http://www.ncbi.nlm.nih.gov/geo). Zinc finger protein family genes were obtained from the UniProt database (https://www.uniprot.org/), with the full list provided in the Supplementary Materials. All R scripts and associated data used to construct and validate the ZNF-based prognostic model, including DEG analysis, Cox regression, GSEA, immune infiltration, immunotherapy prediction, drug sensitivity, and TMB analyses, have been deposited in Zenodo and are publicly available via DOI: https://doi.org/10.5281/zenodo.17263861.

## Supplementary Materials

The following supporting information can be downloaded at: https://www.mdpi.com/article/10.3390/cancers18101591/s1, Supplementary Table S1: Zinc finger protein family genes from the UniProt database; Supplementary Table S2: Plasmid sequences for FGD3 overexpression; Supplementary Table S3: SiRNA sequences used for FGD3 knockdown; Supplementary Figure S1: OS curve for low- and high-TMB subgroups in LUAD-TCGA cohort; Supplementary Figure S2: Stratified survival analysis of risk-related model genes in TCGA dataset; Supplementary Figure S3: FGD3 expression in LUAD and enrichment analysis of FGD3-associated DEGs. (**A**) Paired expression of FGD3 in the TCGA dataset. (**B**,**C**) Paired expression of FGD3 in GSE32863 (**B**) and GSE75037 (**C**) datasets. (**D**–**F**) Enrichment analysis of DEGs associated with FGD3: (**D**) GO, (**E**) KEGG, and (**F**) GSEA; Supplementary Figure S4: The IC_50_ of lorlatinib in H3122 and H3122LR cell lines; Supplementary Figure S5: FGD3 knockdown confers lorlatinib resistance in ALK-positive LUAD cells. (**A**,**D**) Western blot validation of FGD3 knockdown efficiency in H3122 (**A**) and H2228 (**D**) cells using specific siRNAs (siControl as negative control). (**B**,**E**) Quantitative analysis of FGD3 protein levels; knockdown efficiency exceeded 70% in both cell lines (unpaired *t*-test). (**C**,**F**) IC_50_ values of lorlatinib in siControl- and siFGD3-treated H3122 (**C**) and H2228 (**F**) cells. FGD3 knockdown significantly increased the IC_50_ of lorlatinib in both cell lines (unpaired *t*-test). Statistical significance: * *p* < 0.05, ** *p* < 0.01, *** *p* < 0.001, **** *p* < 0.0001. Supplementary Original Blot Figure: Uncropped Western blot images. The uncropped blots for Figure 11A,F and Figure 12A, and Supplementary Figure S5A,D are shown in this file.

## Abbreviations

The following abbreviations are used in this manuscript:

AUC	Area under the curve
BP	Biological process
CC	Cellular component
CCK-8	Cell Counting Kit-8
CI	Confidence interval
CML	Chronic myeloid leukemia
CNV	Copy number variation
CTRP	Cancer Therapeutics Response Portal
DAB	3,3′-Diaminobenzidine
DE-ZNF	Differentially expressed ZNF
DEG	Differentially expressed gene
FBS	Fetal bovine serum
FDR	False discovery rate
GDSC	Genomics of Drug Sensitivity in Cancer
GEO	Gene Expression Omnibus
GO	Gene Ontology
GSEA	Gene set enrichment analysis
GSVA	Gene set variation analysis
H3122LR	H3122 lorlatinib-resistant cells
HBE	Human bronchial epithelial
HPA	Human Protein Atlas
HR	Hazard ratio
IC50	Half-maximal inhibitory concentration
ICB	Immune checkpoint blockade
ICI	Immune checkpoint inhibitor
IHC	Immunohistochemistry
IPS	Immunophenoscore
IRS	Immunoreactive score
KEGG	Kyoto Encyclopedia of Genes and Genomes
LASSO	Least absolute shrinkage and selection operator
LUAD	Lung adenocarcinoma
MF	Molecular function
NSCLC	Non-small-cell lung cancer
OS	Overall survival
PVDF	Polyvinylidene fluoride
ROC	Receiver operating characteristic
ssGSEA	Single-sample gene set enrichment analysis
STR	Short tandem repeat
TCGA	The Cancer Genome Atlas
TIDE	Tumor immune dysfunction and exclusion
TMB	Tumor mutation burden
UniProt	Universal Protein Resource
ZNF	Zinc finger protein
