# Supplementary material for: A Zinc Finger Protein-Based Prognostic Model in Lung Adenocarcinoma Identifies FGD3 as a Marker Associated with Lorlatinib Resistance
Source: Cancers (Basel). 2026 May 14;18(10):1591. doi: 10.3390/cancers18101591 (PMC13205127; doi:10.3390/cancers18101591)
Supplement: Supplementary file 1 [file cancers-18-01591-s001.zip › Supplementary Captions.pdf]

Supplementary Table Captions

Supplementary Table S1
Zinc finger protein family genes from the Universal Protein Resource database.
Supplementary Table S2
Plasmid sequences for FGD3 overexpression.
Supplementary Table S3
SiRNA sequences used for FGD3 knockdown.

Supplementary Figure Captions

Supplementary Figure S1
OS survival curve for low- and high-TMB subgroups in LUAD-TCGA cohort.
Supplementary Figure S2
Stratified survival analysis of risk-related model genes in TCGA dataset.
Supplementary Figure S3
FGD3 expression in LUAD and enrichment analysis of FGD3 associated differentially expressed genes. (A) Paired expression of FGD3 in the TCGA dataset. (B – C) Paired expression of FGD3 in GSE32863 (B) and GSE75037 (C) datasets. (D – F) Enrichment analysis of differentially expressed genes associated with FGD3: (D) GO, (E) KEGG, and (F) GSEA.
Supplementary Figure S4
The IC_{50} of lorlatinib in H3122 and H3122LR cell lines.
Supplementary Figure S5
FGD3 knockdown confers lorlatinib resistance in ALK-positive LUAD cells. (A, D) Western blot validation of FGD3 knockdown efficiency in H3122 (A) and H2228 (D) cells using specific siRNAs (siControl as negative control). (B, E) Quantitative analysis of FGD3 protein levels; knockdown efficiency exceeded 70% in both cell lines (unpaired t-test). (C, F) IC_{50} values of lorlatinib in siControl-and siFGD3-treated H3122 (C) and H2228 (F) cells. FGD3 knockdown significantly increased the IC_{50} of lorlatinib in both cell lines (unpaired t-test). Statistical significance: *$P < 0.05$, **$P < 0.01$, ***$P < 0.001$, ****$P < 0.0001$.
