# Supplementary figures and images for: A Zinc Finger Protein-Based Prognostic Model in Lung Adenocarcinoma Identifies FGD3 as a Marker Associated with Lorlatinib Resistance
Source: Cancers (Basel). 2026 May 14;18(10):1591. doi: 10.3390/cancers18101591 (PMC13205127; doi:10.3390/cancers18101591)

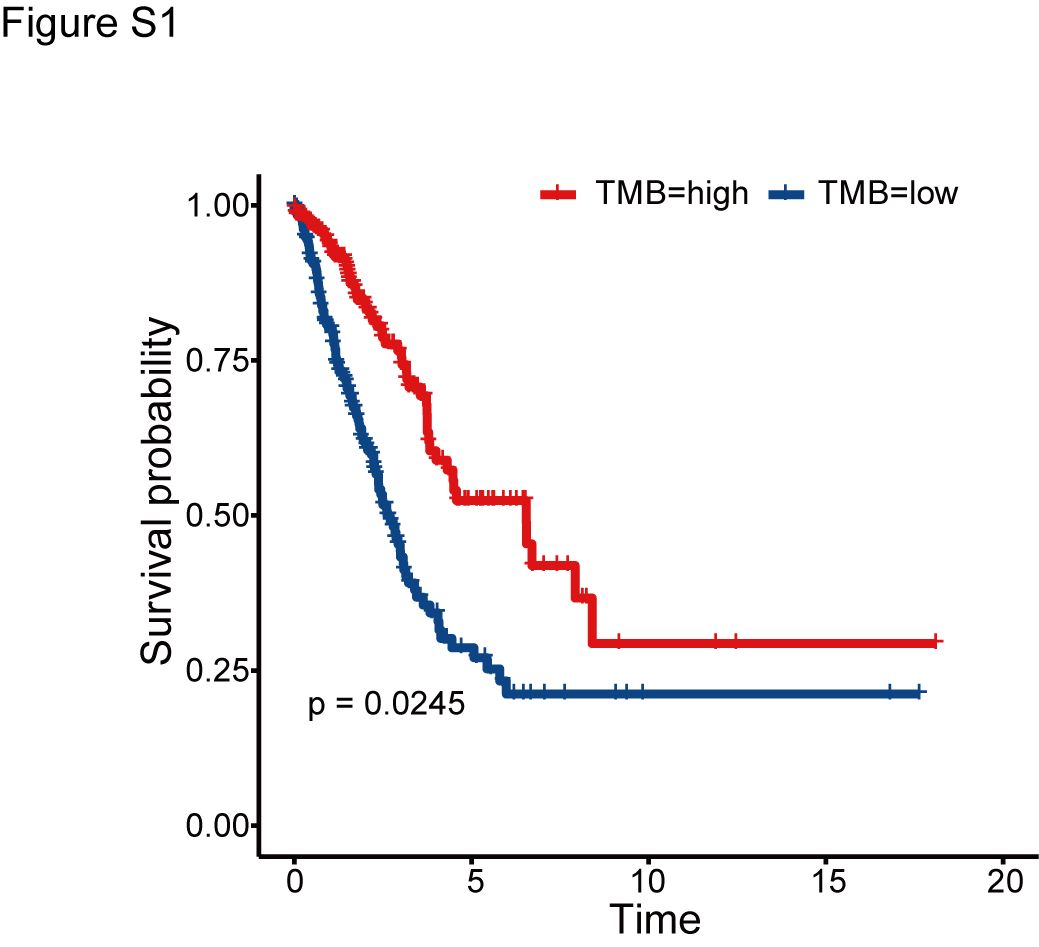

Supplement: Supplementary file 1 [file cancers-18-01591-s001.zip › FigureS1.tif]

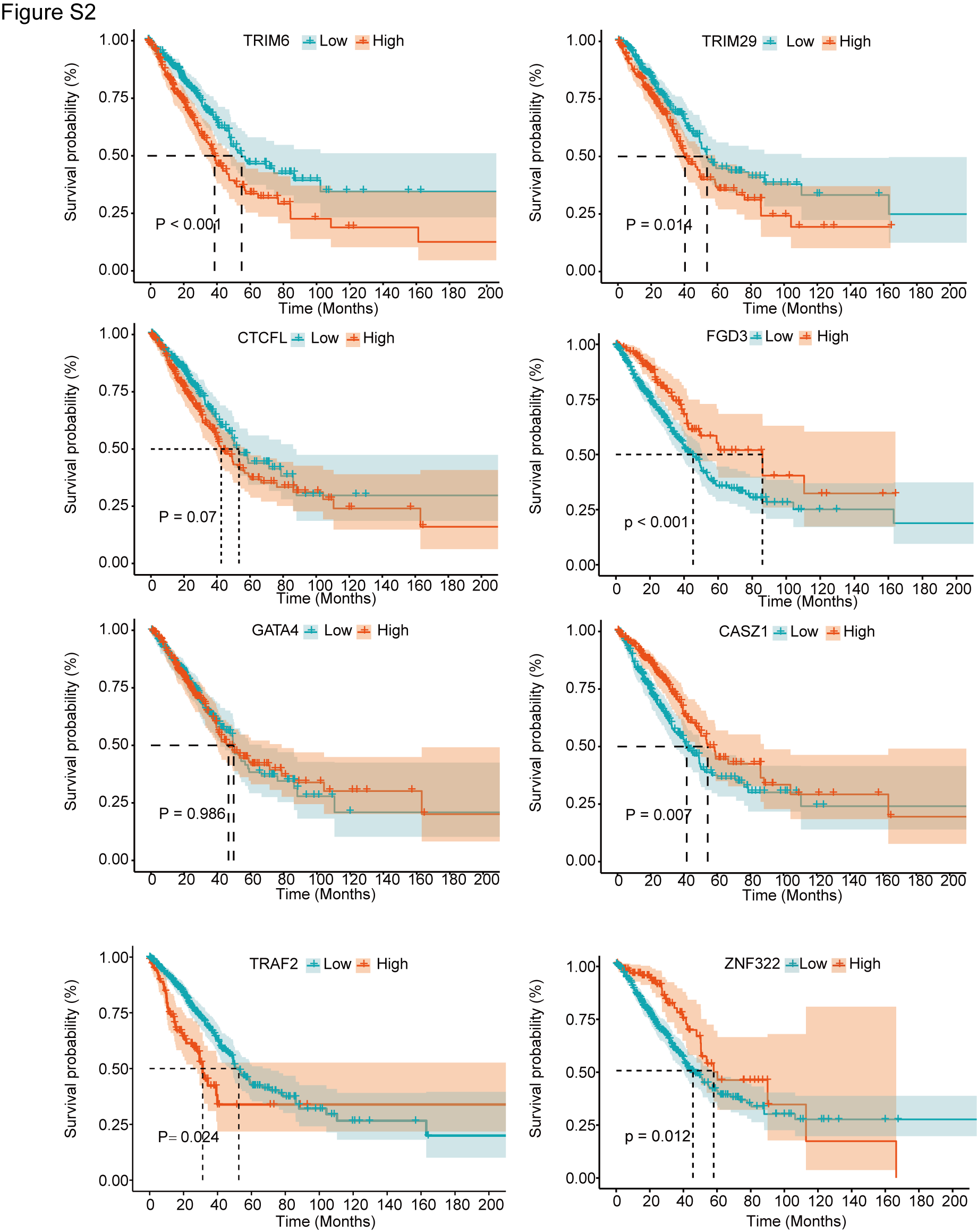

Supplement: Supplementary file 1 [file cancers-18-01591-s001.zip › FigureS2.tif]

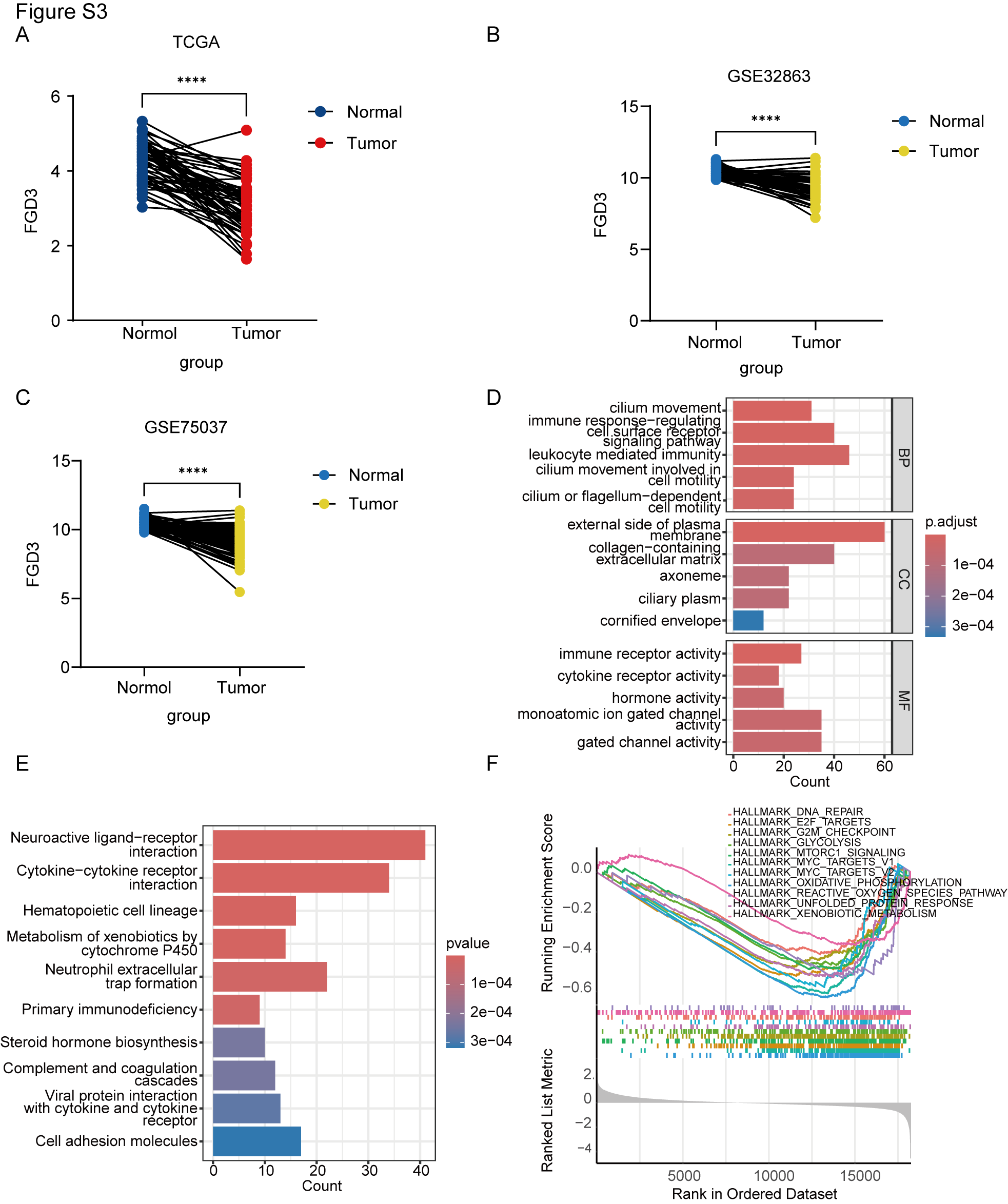

Supplement: Supplementary file 1 [file cancers-18-01591-s001.zip › FigureS3.tif]

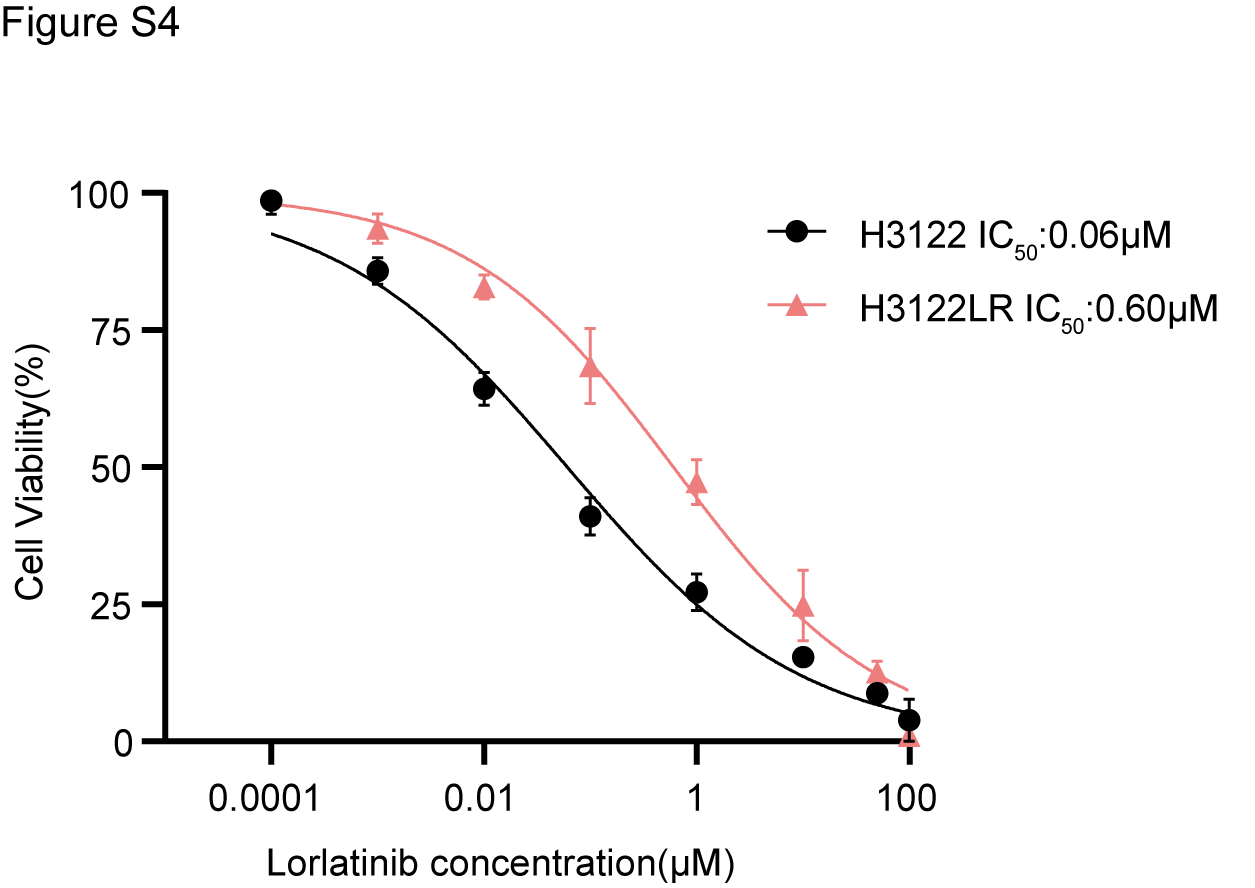

Supplement: Supplementary file 1 [file cancers-18-01591-s001.zip › FigureS4.tif]

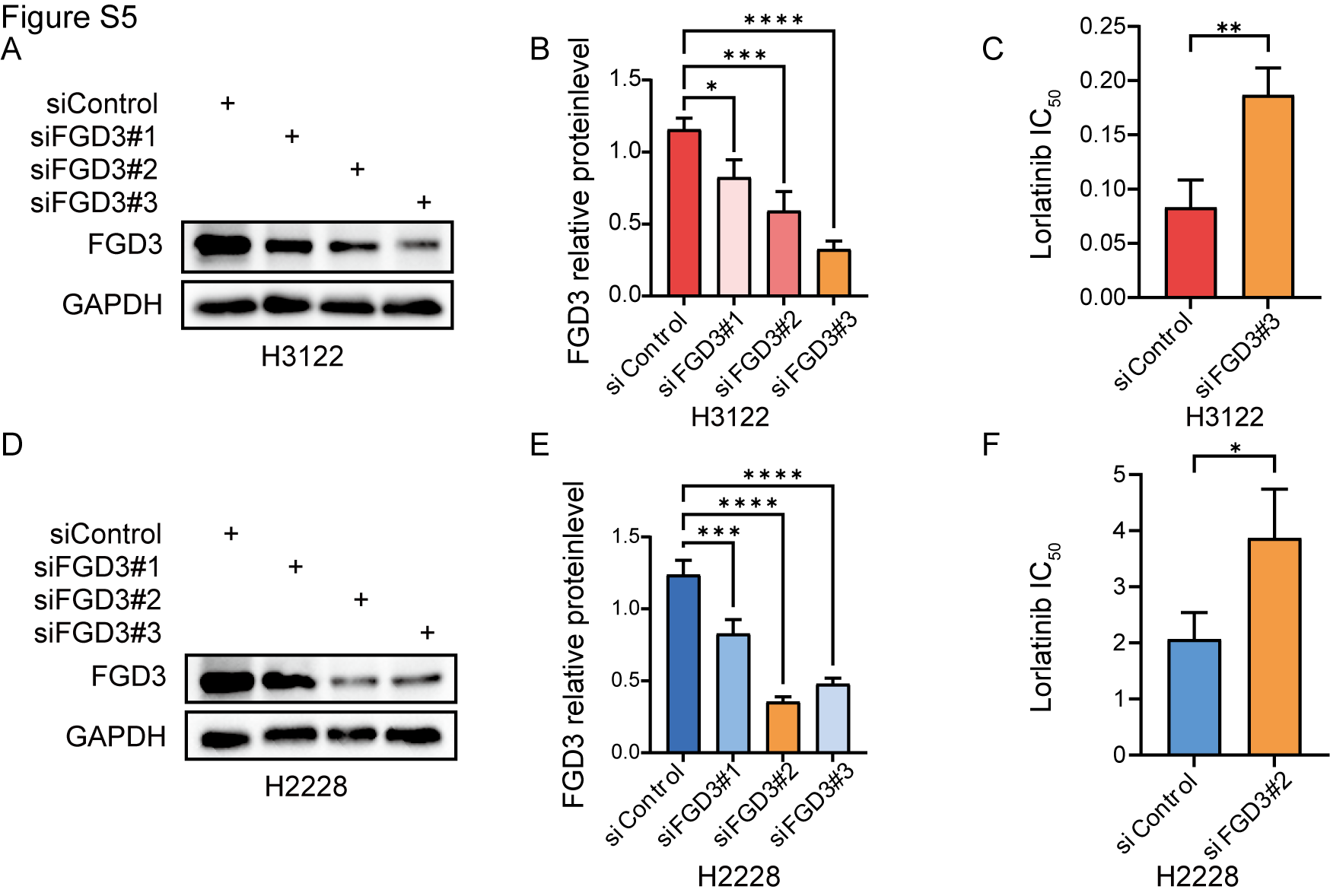

Supplement: Supplementary file 1 [file cancers-18-01591-s001.zip › FigureS5.tif]

## WB

FIGURE11A

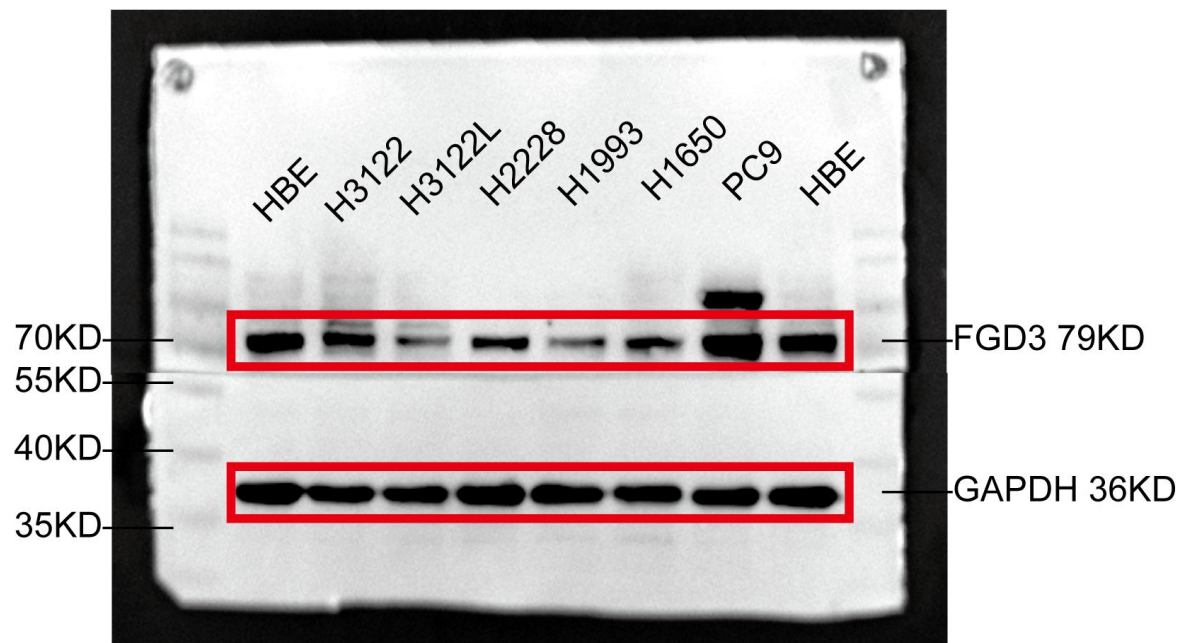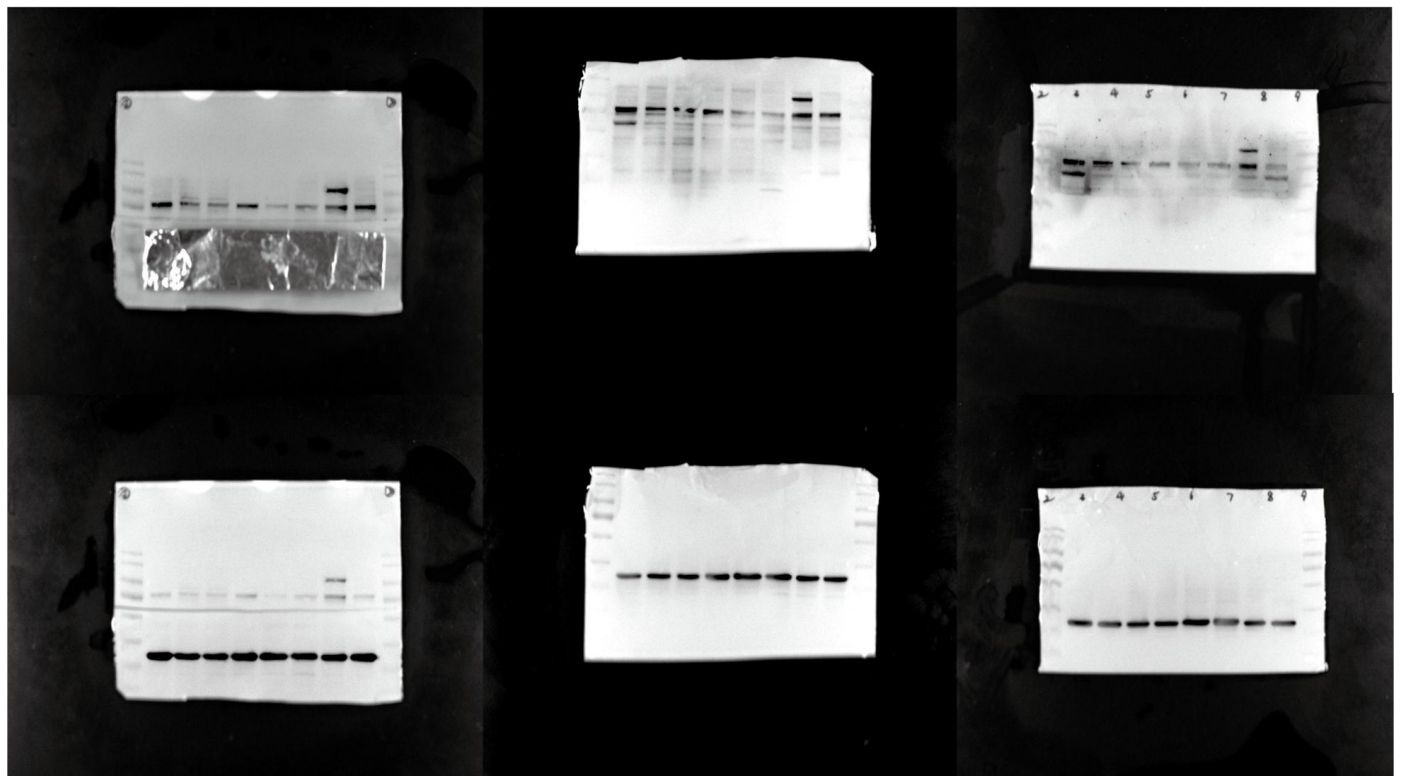

FIGURE11B

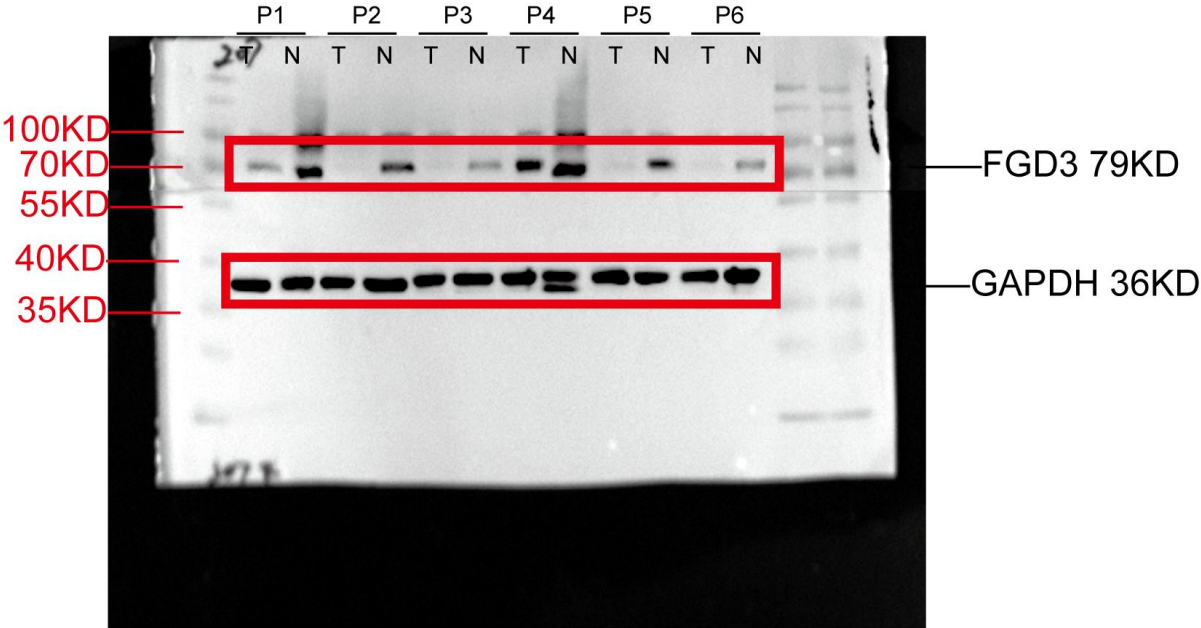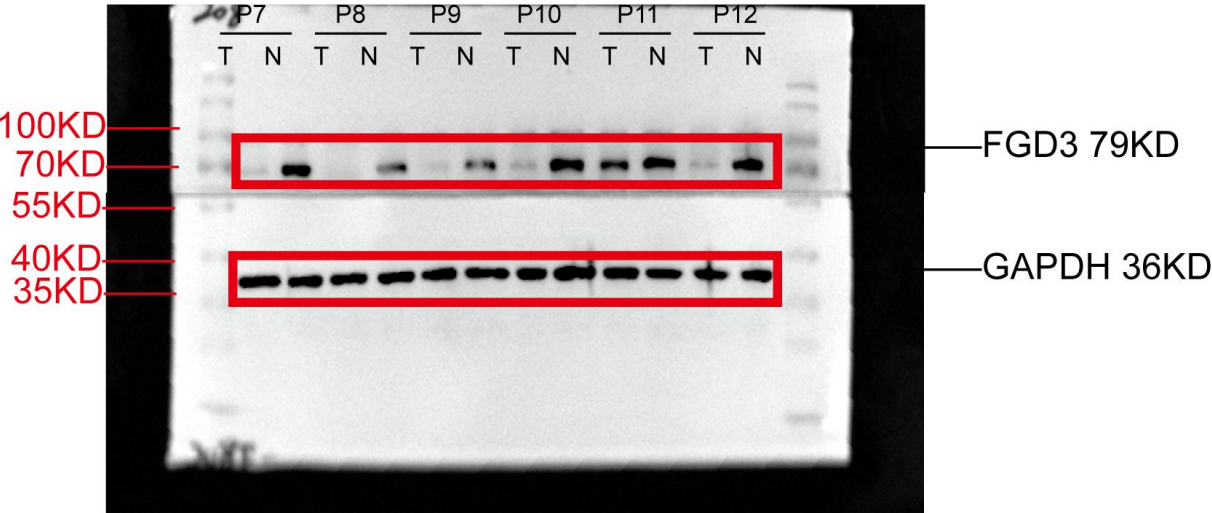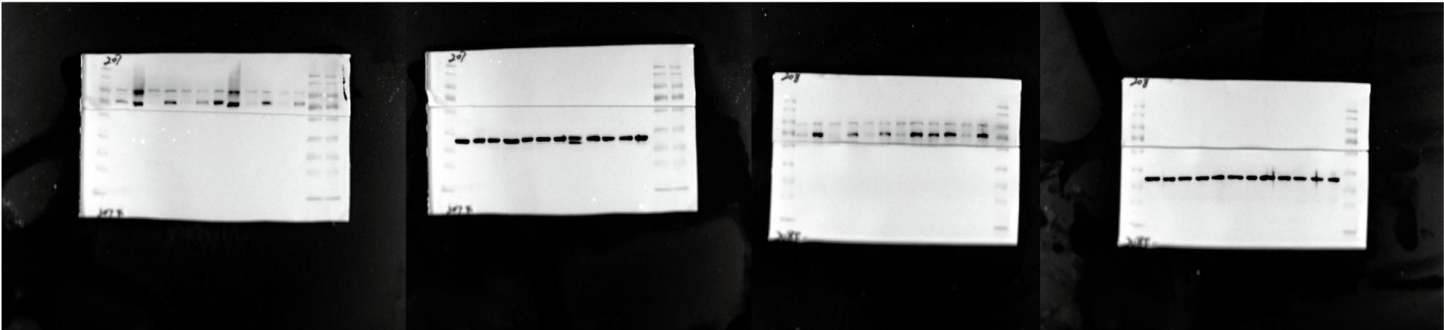

FIGURE11F

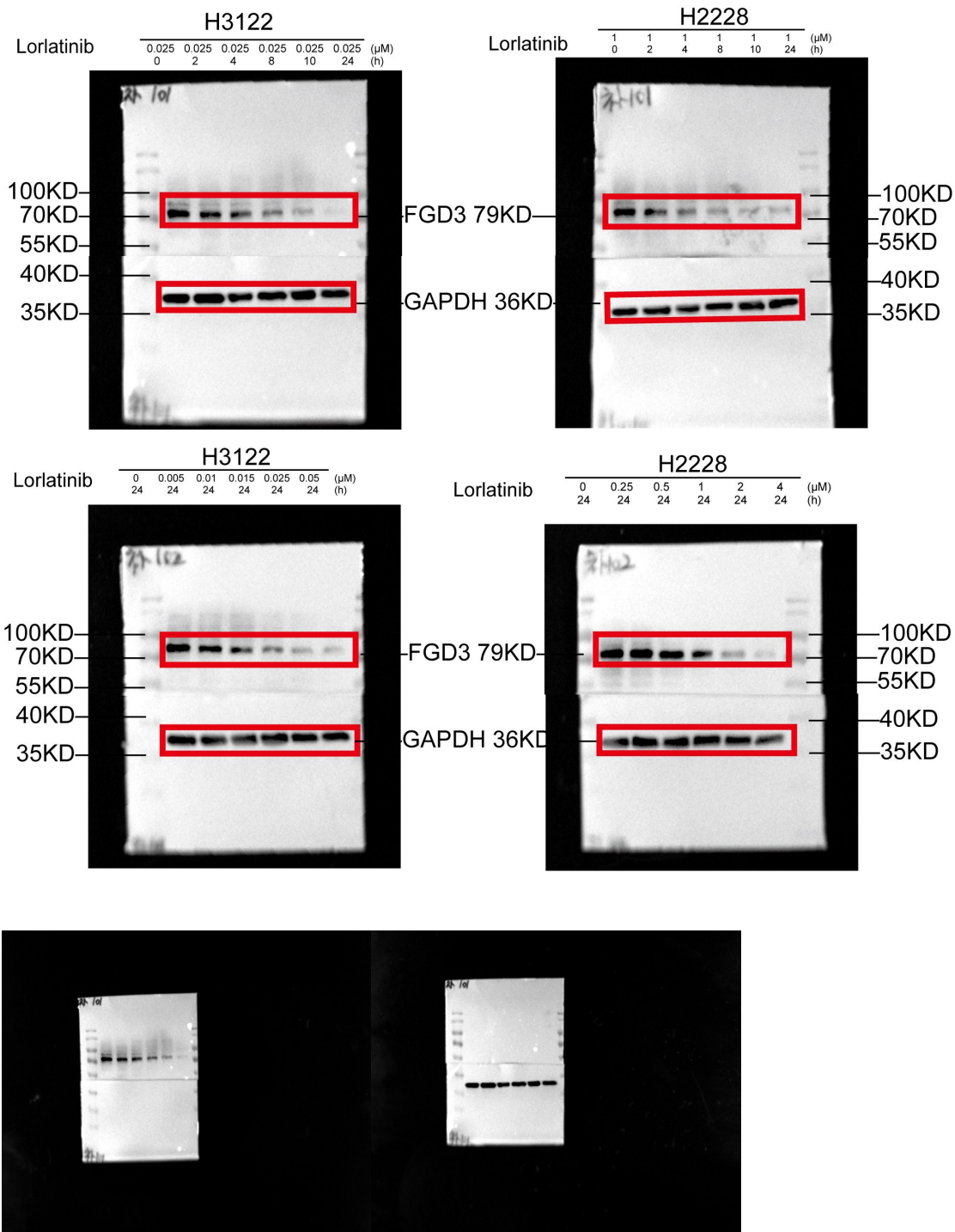

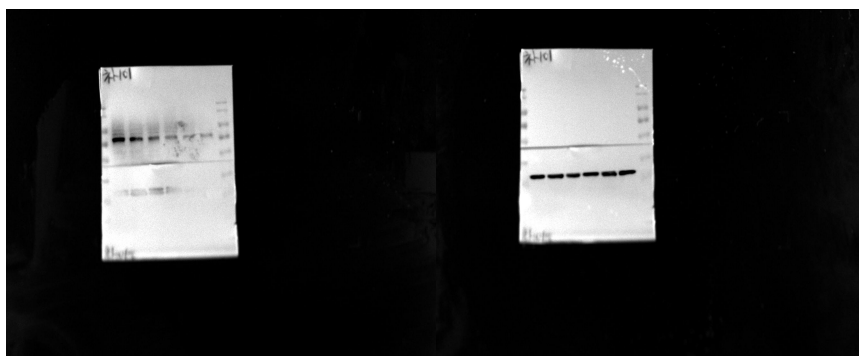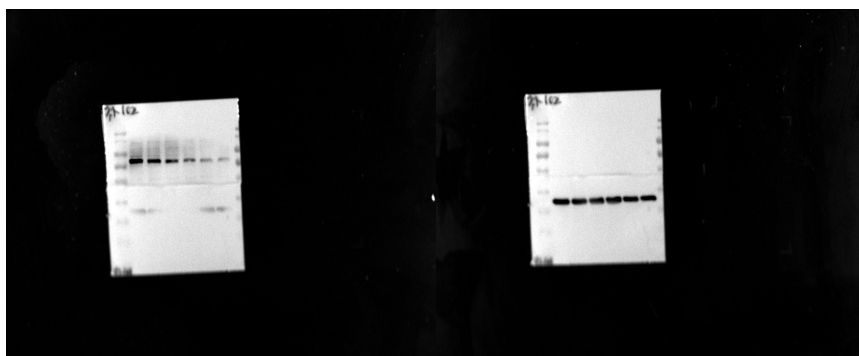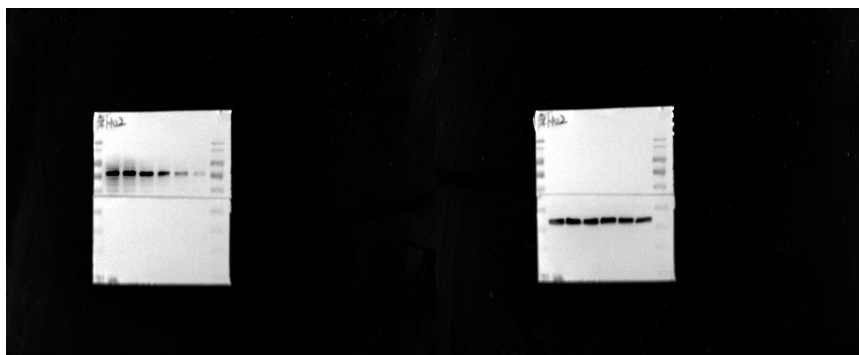

FIGURE 12A  
FGD3

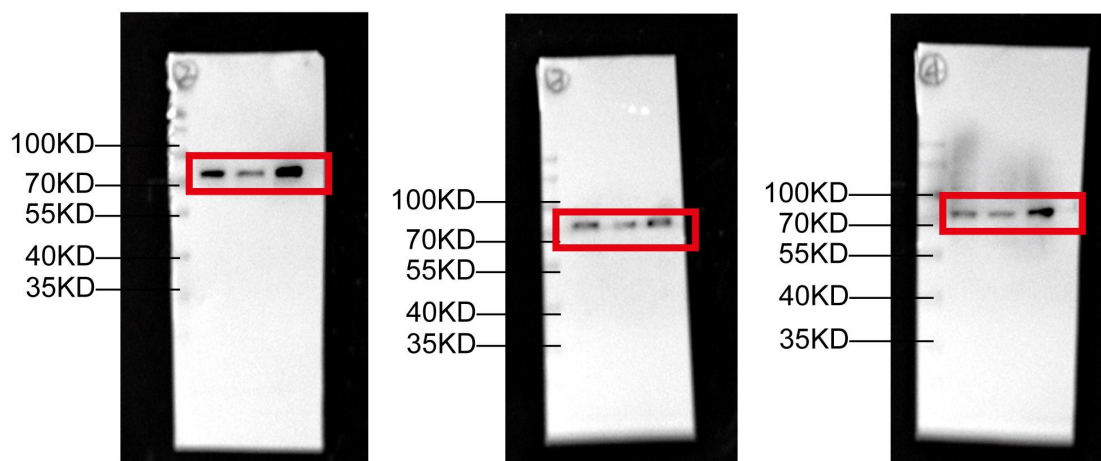

GAPDH

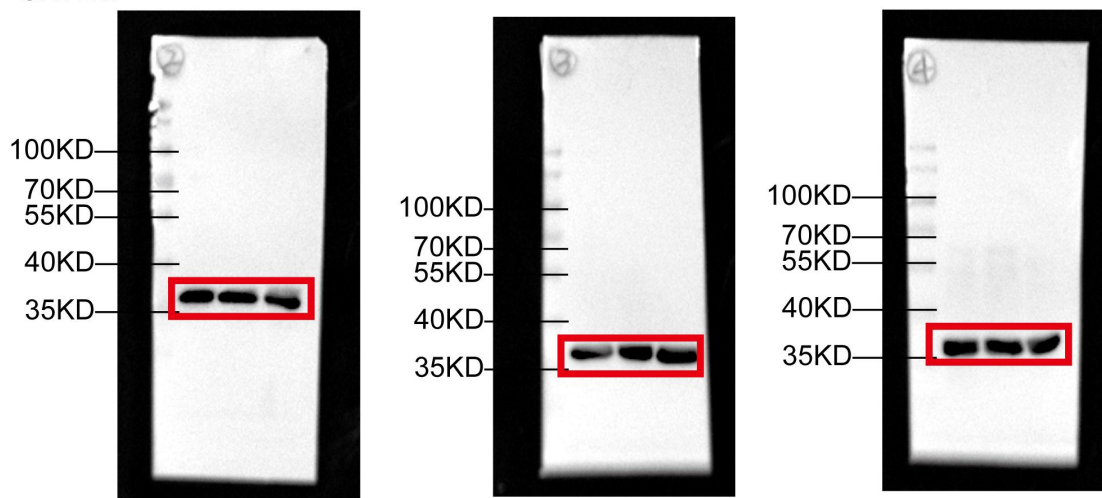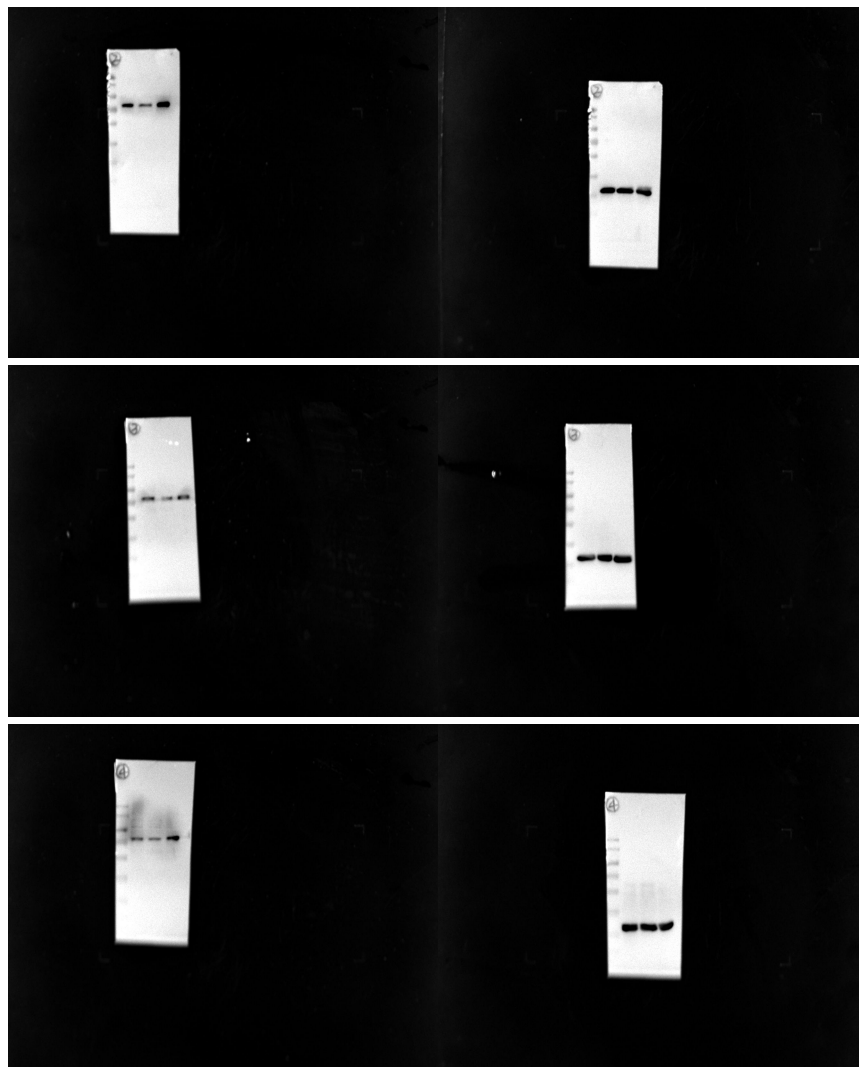

FIGURES5A

FGD3

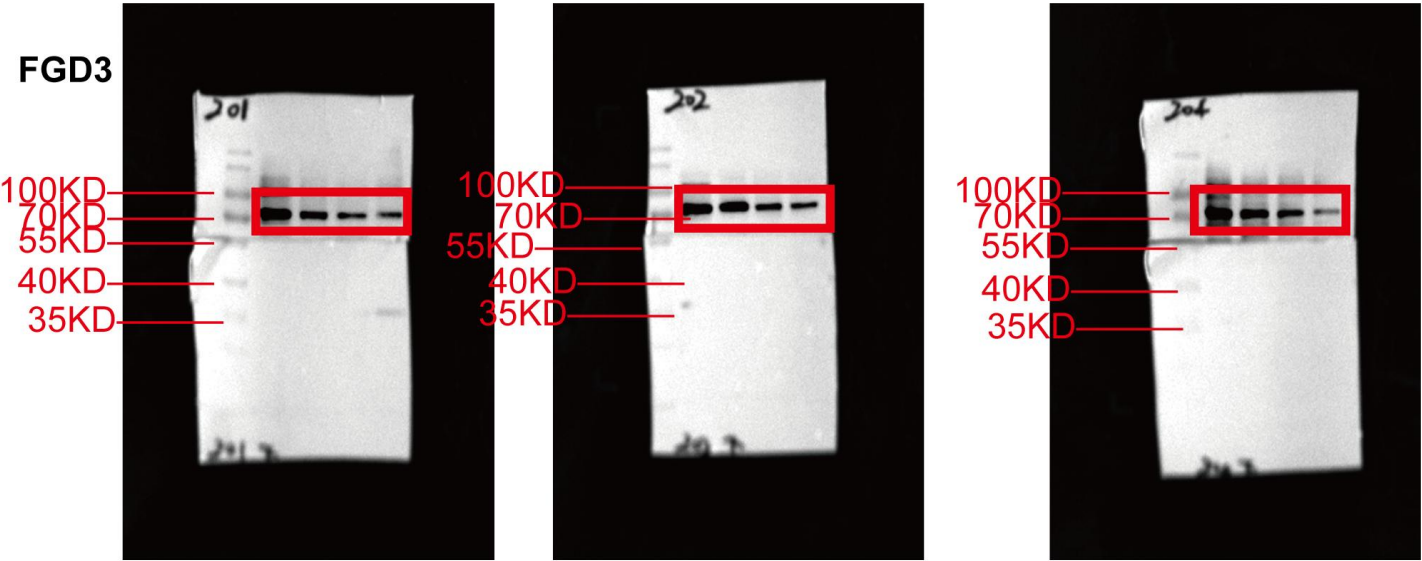

GAPDH

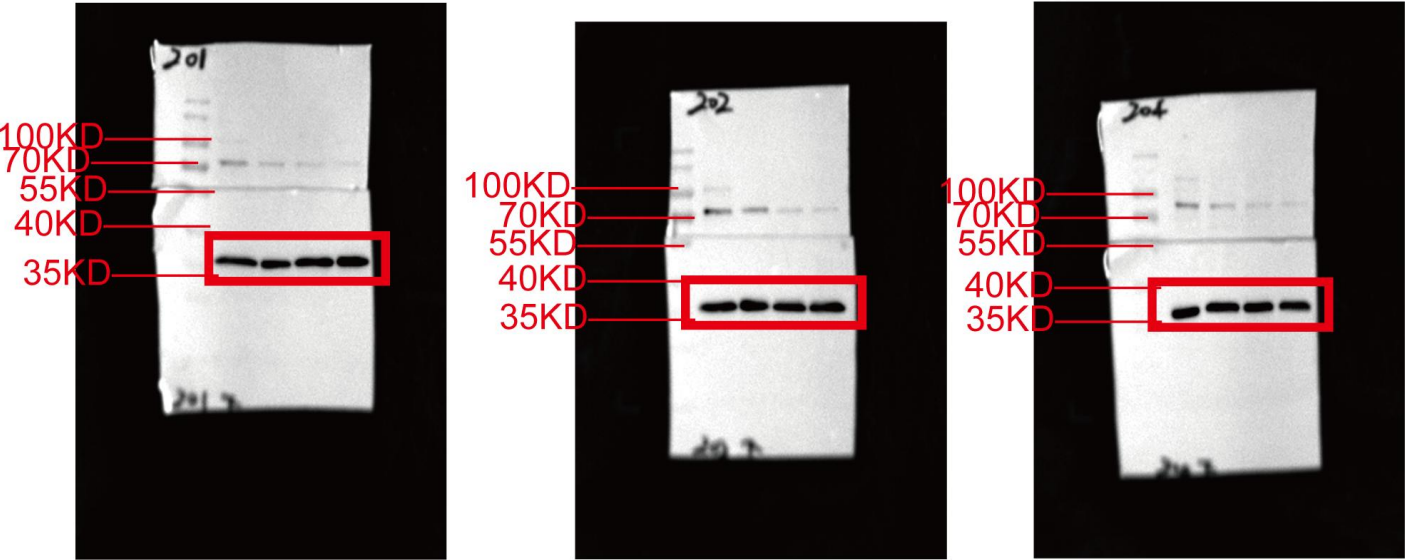

FIGURES 5 D

FGD3

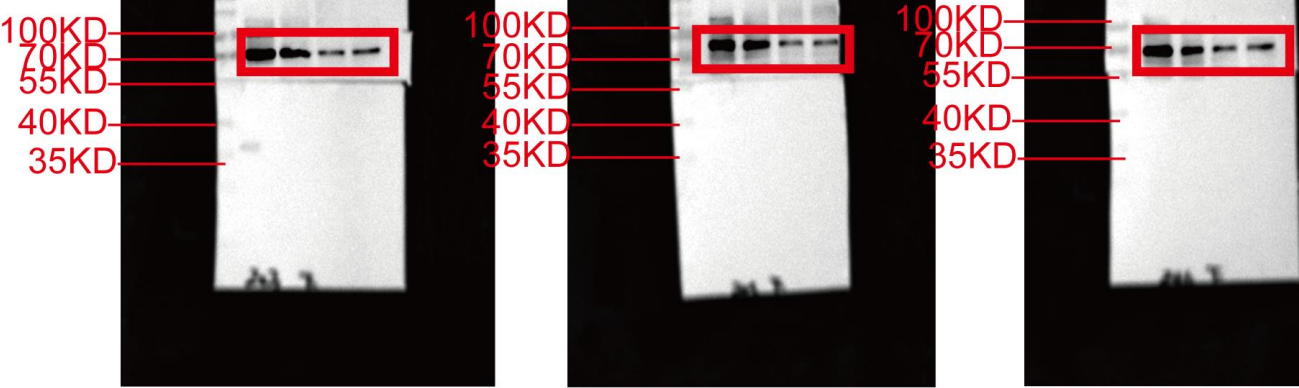

GAPDH

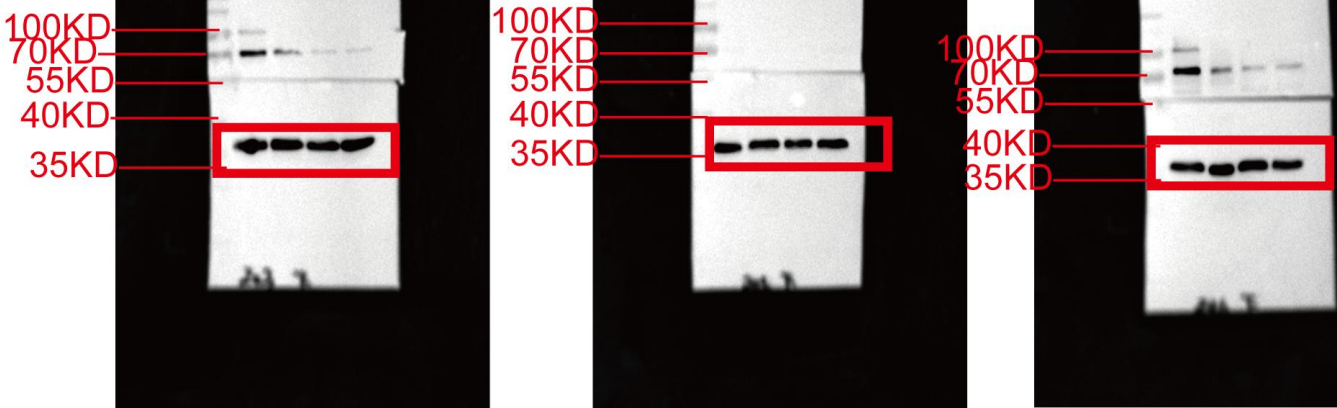

Supplement: Supplementary file 1 [file cancers-18-01591-s001.zip › Supplementary Original Blot Figure.pdf]
